# The WalRK Two-Component System Is Essential for Proper Cell Envelope Biogenesis in Clostridioides difficile

**DOI:** 10.1128/jb.00121-22

**Published:** 2022-05-16

**Authors:** Ute Müh, Craig D. Ellermeier, David S. Weiss

**Affiliations:** a Department of Microbiology and Immunology, University of Iowa, Iowa City, Iowa, USA; University of Chicago

**Keywords:** cell envelope, regulation of gene expression, signal transduction

## Abstract

The WalR-WalK two-component regulatory system (TCS) is found in all *Firmicutes*, in which it regulates the expression of multiple genes required for remodeling the cell envelope during growth and division. Unlike most TCSs, WalRK is essential for viability, so it has attracted interest as a potential antibiotic target. In this study, we used overexpression of WalR and CRISPR interference to investigate the Wal system of Clostridioides difficile, a major cause of hospital-associated diarrhea in high-income countries. We confirmed that the *wal* operon is essential and identified morphological defects and cell lysis as the major terminal phenotypes of altered *wal* expression. We also used transcriptome sequencing (RNA-seq) to identify over 150 genes whose expression changes in response to WalR levels. This gene set is enriched in cell envelope genes and includes genes encoding several predicted PG hydrolases and proteins that could regulate PG hydrolase activity. A distinct feature of the C. difficile cell envelope is the presence of an S-layer, and we found that WalR affects expression of several genes which encode S-layer proteins. An unexpected finding was that some Wal-associated phenotypic defects were inverted in comparison to what has been reported for other *Firmicutes*. For example, downregulation of Wal signaling caused C. difficile cells to become longer rather than shorter, as in Bacillus subtilis. Likewise, downregulation of Wal rendered C. difficile more sensitive to vancomycin, whereas reduced Wal activity is linked to increased vancomycin resistance in Staphylococcus aureus.

**IMPORTANCE** The WalRK two-component system (TCS) is essential for coordinating synthesis and turnover of peptidoglycan in *Firmicutes*. We investigated the WalRK TCS in Clostridioides difficile, an important bacterial pathogen with an atypical cell envelope. We confirmed that WalRK is essential and regulates cell envelope biogenesis, although several of the phenotypic changes we observed were opposite to what has been reported for other *Firmicutes*. We also identified over 150 genes whose expression is controlled either directly or indirectly by WalR. Overall, our findings provide a foundation for future investigations of an important regulatory system and potential antibiotic target in C. difficile.

## INTRODUCTION

To grow and divide, bacteria must thoroughly remodel their peptidoglycan (PG) sacculus, a large, covalently closed macromolecule that surrounds the cell and provides protection against rupture due to turgor pressure (1). How bacteria remodel the sacculus without making errors that lead to inadvertent lysis is a question that has fascinated microbiologists for decades. The question is also of practical significance because small molecules that undermine normal peptidoglycan biogenesis are among our most effective antibiotics.

An important advance in our understanding of cell envelope biogenesis was made over 20 years ago when Fabret and Hoch identified a Bacillus subtilis two-component system (TCS) that is essential for viability (2). This TCS is now called the Wal system and is known to coordinate expression of multiple cell envelope genes in the *Firmicutes*. WalRK is essential in all species studied so far, including pathogens such as Staphylococcus aureus (3–5) and Streptococcus pneumoniae (6, 7). Because of its essentiality, the Wal TCS has attracted interest as a potential antibiotic target.

The Wal TCS comprises the bifunctional kinase/phosphatase WalK together with its cognate response regulator, WalR. In addition, *wal* operons typically include genes for membrane proteins known or presumed to modulate WalK activity, but the accessory proteins differ between organisms (8–10). Recent evidence from B. subtilis indicates that WalK activity is regulated by PG fragments generated by PG hydrolases that open spaces to make room for elongation. These PG fragments bind to WalK’s extracellular Cache domain to modulate the phosphorylation state of WalR (11). Phosphorylated WalR, in turn, binds to various promoters to activate or repress gene expression.

The number of genes in the WalR regulon ranges from about a dozen to over 100 (5, 12–16), depending on the species, and only some of these genes are directly activated or repressed by WalR. Although WalR regulons are diverse, they invariably include multiple proteins that contribute to proper biogenesis of the cell envelope, especially PG hydrolases. Consistent with these themes, the phenotypic defects elicited by artificial up- or downregulation of WalRK signaling include abnormal cell shape, larger cells, smaller cells, ghost cells (lysis), thicker PG, abnormal division septa, and altered sensitivity to antibiotics that target PG biogenesis (8–10). In S. pneumoniae *walRK* is essential because it activates expression of an essential PG hydrolase gene, *pcsB* (6, 7). However, the essentiality of *walRK* in B. subtilis and S. aureus is polygenic in nature, resulting from abnormal expression of multiple genes that are not by themselves essential. In all three organisms, it is possible to bypass the *walRK* requirement by constitutively expressing one or more Wal regulon PG hydrolase genes, sometimes in combination with deleting genes for hydrolase inhibitors (6, 17, 18).

**FIG 1:**
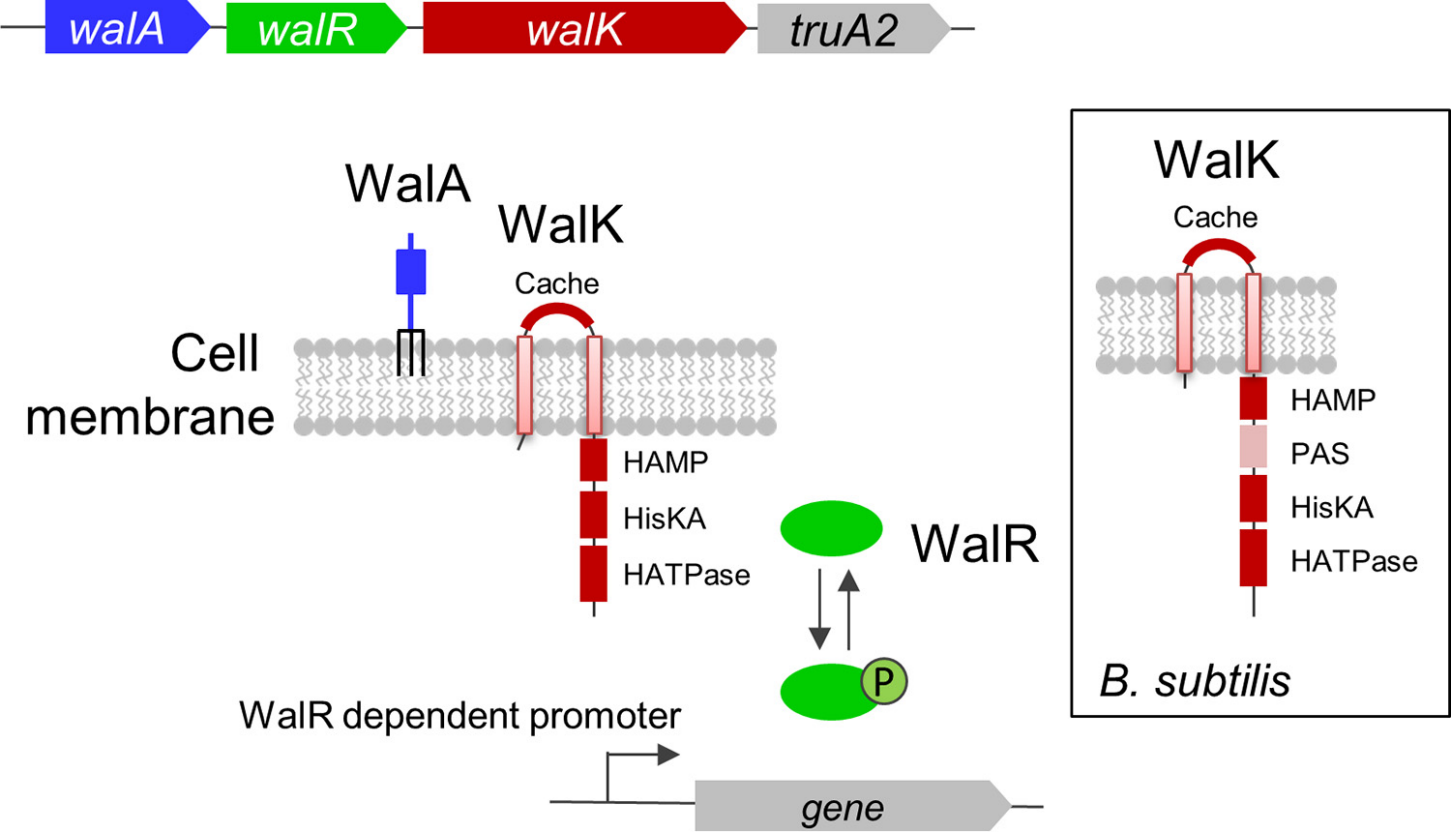
The *wal* operon of C. difficile. Based on studies in other bacteria, WalK (red) is a bifunctional signal-transducing enzyme that can phosphorylate or dephosphorylate WalR (green). WalK is predicted to have an extracellular Cache domain, as well as intracellular HAMP, HisKA (phospho-acceptor), and HATPase domains. Unlike the B. subtilis ortholog (inset), it does not have an intracellular PAS domain. WalR~P binds DNA to activate or repress expression of Wal-regulated genes. The lipoprotein (WalA [blue]), which is unique to C. difficile, may modulate WalK activity and is predicted to have a beta-propeller lyase domain. The *truA2* gene codes for a pseudouridylate synthase and is not thought to play a role in Wal signaling. The operon locus is *cdr20291_1676-1679* in R20291 and *cd630_17810-17840* in 630Δ*erm*.

Here, we studied the WalRK TCS in Clostridioides difficile, an anaerobic, spore-forming member of the *Firmicutes* responsible for close to a quarter of a million hospitalizations and over 12,000 deaths per year in the United States (19). Unlike the envelope of most *Firmicutes* in which *walRK* has been studied previously, the C. difficile envelope has a proteinaceous S-layer whose assembly presumably must be coordinated with PG synthesis (20–22). In addition, the PG itself is somewhat unusual (23). About 90% of the *N*-acetylglucosamine (GlcNAc) is deacetylated (which has implications for PG hydrolase activity), and about 70% of the peptide cross-links are 3-3 rather than 4-3 (which has implications for the structure of PG fragments thought to bind to the Cache domain of WalK [11]). The C. difficile Wal system includes a unique lipoprotein gene and a noncanonical WalK that lacks an intracellular PER-ARNT-SIM (PAS) signaling domain (Fig. 1) (10). These features distinguish C. difficile’s WalRK TCS from those of previously characterized *Firmicutes*, although a distantly related *wal*-like system in Mycobacterium tuberculosis (MtrAB) includes a lipoprotein and a WalK-like histidine kinase without an intracellular PAS domain (10, 24, 25). Finally, our recent development of tools for xylose-inducible gene expression and CRISPR interference (26) helped to overcome some of the challenges inherent in phenotypic analysis of essential genes like *walRK* in C. difficile.

## RESULTS

### The *wal* operon is required for cell viability, proper rod morphology, and intrinsic resistance to some antibiotics.

According to transposon insertion sequencing (Tn-seq), the WalRK TCS is essential in C. difficile (27). To confirm and extend this finding, we leveraged the power of CRISPR interference (CRISPRi) for functional analysis of essential genes/operons (28). Our CRISPRi system comprises a xylose-inducible nuclease-defective Cas9, P*_xyl_*::*dCas9*, that is targeted to a gene of interest by a single guide RNA (sgRNA) expressed constitutively from the glutamate dehydrogenase promoter, P*_gdh_*::*sgRNA* (26). Because CRISPRi is polar, we decided to target the first gene in the operon, which encodes a predicted lipoprotein unique to the C. difficile *wal* operon (Fig. 1). We have named this gene *walA*. For reproducibility, we designed two guides against *walA*, sgRNA-*walA1* and sgRNA-*walA2*. Subsequent transcriptome sequencing (RNA-seq) experiments described below confirmed that targeting dCas9 to *walA* suppressed transcription of the entire operon. As a negative control, we used an sgRNA (sgRNA-neg) that does not have a target anywhere on the C. difficile chromosome. In some experiments the CRISPRi machinery was produced from a plasmid that confers resistance to thiamphenicol (Thi), while in others it was integrated into the chromosome at the *pyrE* locus in a way that does not leave behind an antibiotic resistance marker (29).

**FIG 2:**
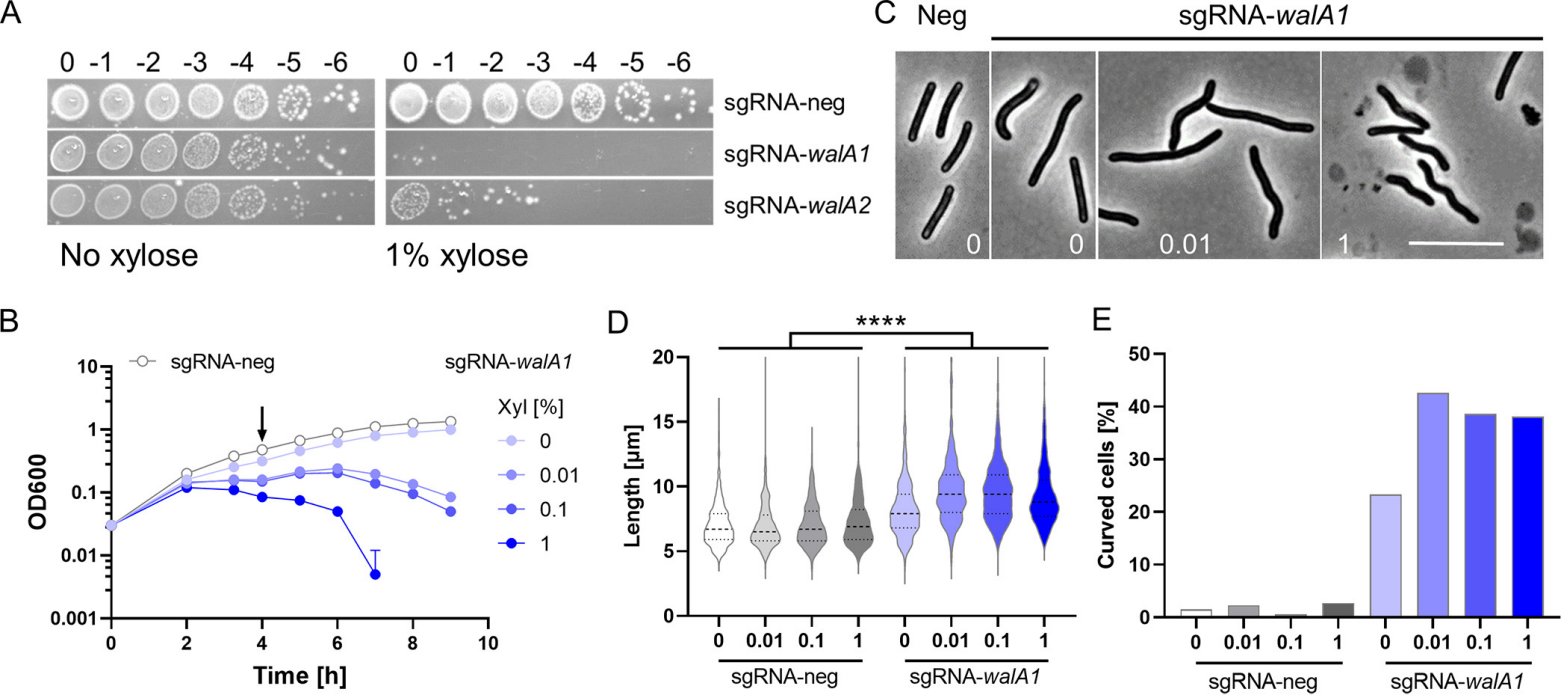
The *wal* operon is required for viability and normal rod morphology of C. difficile 630Δ*erm*. (A) Viability assay. Serial dilutions of overnight cultures were spotted onto TY plates with or without 1% xylose to induce the expression of *dCas9*. Plates were photographed after incubation overnight. Strains carry chromosomal copies of the CRISPRi components and the following guides: sgRNA-neg (UM554), sgRNA-*walA1* (UM555), and sgRNA-*walA2* (UM556). (B) Growth curves in liquid TY containing 0, 0.01, 0.1, or 1% xylose as indicated. For clarity, the sgRNA-neg control strain is graphed only at 0% xylose. Samples were taken at 4 h (arrow) for phase-contrast microscopy (C), cell length measurements (D), and determination of curvature (E). Numbers at the bottom of micrographs in panel C refer to percent xylose. Note cell debris indicative of lysis at 1% xylose. Bar = 10 μm. Cell length and percent curved cells were based on about 500 cells per condition. The sgRNA-*walA1* strain is longer than the sgRNA-neg control in all pairwise comparisons as determined by a *t* test (*P* < 0.0001).

Chromosomal- or plasmid-based CRISPRi knockdown of the *wal* operon in either 630Δ*erm* or R20291 reduced viability ≥10^4^-fold when cells were plated on tryptone-yeast extract (TY) agar containing 1% xylose (Fig. 2A; see also Fig. S1A in the supplemental material). Additional phenotypes were characterized in TY broth using 630Δ*erm* derivatives with the CRISPRi machinery integrated at *pyrE*. In the absence of xylose, sgRNA-*walA1* and sgRNA-*walA2* retarded growth slightly compared to the sgRNA-neg control (Fig. 2B and Fig. S1B). This finding indicates that there is leaky expression of P*_xyl_*::*dCas9*, which was later confirmed by RNA-seq (see below). Under these conditions, cells were, on average, about 10% longer than the wild type and the fraction of cells with curved or irregular contours increased from ~1% in the sgRNA-neg control to ~20% for sgRNA-*walA1* or ~10% for sgRNA-*walA2* (Fig. 2C to E and Fig. S1E and F). Further knockdown of the *wal* operon by addition of xylose exacerbated the growth defect in a dose-dependent manner (Fig. 2B and Fig. S1D). In addition, morphological defects became more pronounced, with cells now averaging ~30% longer than controls and up to ~40% exhibiting curved or irregular contours (Fig. 2D and E and Fig. S1E and F).

Partial knockdown of the *wal* operon in the absence of xylose allowed us to screen for altered sensitivity to antibiotics that target cell envelope biogenesis. We found that the MIC for ampicillin and daptomycin decreased about 2-fold, while the MIC for vancomycin decreased about 4-fold (Table 1). But sensitivity to the other two cell wall-active antibiotics, imipenem and bacitracin, and the gyrase inhibitor novobiocin was not affected (Table 1).

**TABLE 1:** Effect of CRISPRi-silencing *wal* on sensitivity to antibiotics^*a*^

Antibiotic	MIC (μg/mL)		
	sgRNA-neg	sgRNA-*walA1*	sgRNA-*walA2*
Ampicillin	6.3	3.5	3.1
Imipenem	3.1	3.1	2.6
Vancomycin	4.6	0.9	0.7
Daptomycin	11.3	4.1	5.7
Bacitracin	250	250	250
Novobiocin	13	17	16

a Results are the averages of measurements done in duplicate on four different days.

### Overexpression of WalR impairs growth, alters morphology, and slows autolysis.

**FIG 3:**
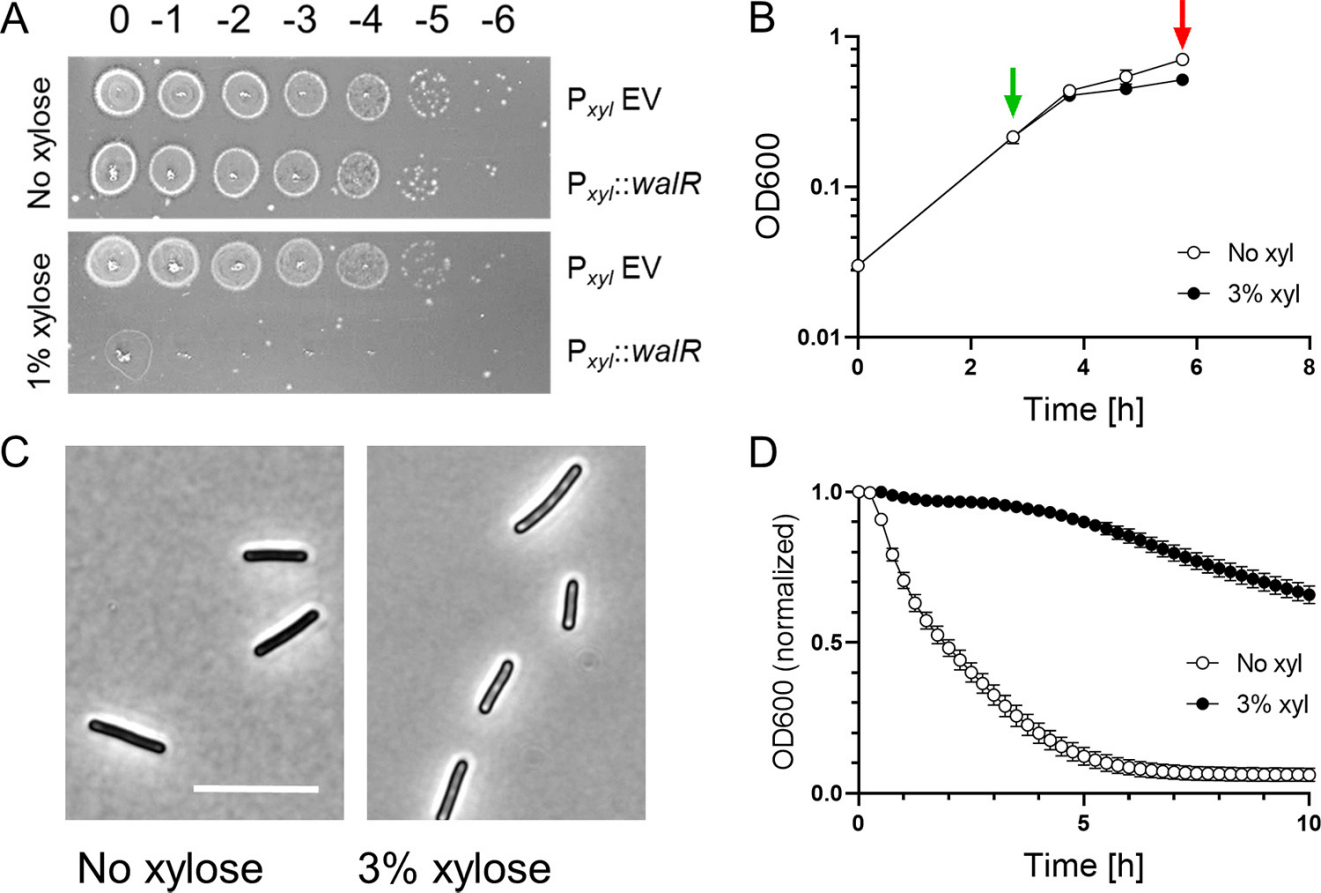
Overexpression of *walR* from a multicopy plasmid impairs growth, alters morphology, and slows autolysis in 630Δ*erm*. (A) Viability assay. Overnight cultures of 630Δ*erm* harboring pCE691 (P*_xyl_*::*walR*) or the empty vector control plasmid pBZ101 (P*_xyl_* EV) were serially diluted and spotted onto TY-Thi plates with or without 1% xylose. Plates were photographed after incubation overnight. (B) Growth curve of 630Δ*erm*/pCE691 (P*_xyl_*::*walR*). Duplicate cultures were grown to an OD_600_ of 0.2, at which time one was induced with 3% xylose (green arrow). Both cultures were harvested after 3 h (red arrow) for analysis by phase-contrast microscopy (C) and a lysis assay (D). Bar in panel C = 10 μm. For the lysis assay, cells were suspended in buffer containing 0.01% Triton X-100 and OD_600_ was monitored over time. Error bars depict SD of 3 technical replicates. Data shown are representative of results from at least 3 independent experiments.

We also investigated Wal function in C. difficile by overproducing the response regulator WalR to drive cells into an exacerbated Wal-ON state (5, 30). Plating C. difficile 630Δ*erm* harboring a P*_xyl_*::*walR* expression plasmid onto TY-Thi containing 1% xylose resulted in a 10^6^-fold loss of viability (Fig. 3A). This strain failed to grow when subcultured directly into TY-Thi broth containing 1% xylose, but waiting until the optical density at 600 nm (OD_600_) reached ~0.2 before adding 3% xylose resulted in only a subtle growth defect (Fig. 3B). Three hours postinduction, the cells had become strikingly phase bright and resistant to autolysis when suspended in buffer containing 0.01% Triton X-100 (Fig. 3C and D). Triton X-100 is thought to reduce the inhibitory effect of lipoteichoic acids on PG hydrolases (31, 32). Similar results were obtained in the R20291 strain background (Fig. S3). It is unclear what causes cells to turn phase bright upon overexpression of *walR*. Differential staining with Syto 9 and propidium iodide (LIVE/DEAD staining) indicated that >99% of the cells were alive after overproduction of WalR (Fig. 3B, red arrow).

**FIG 4:**
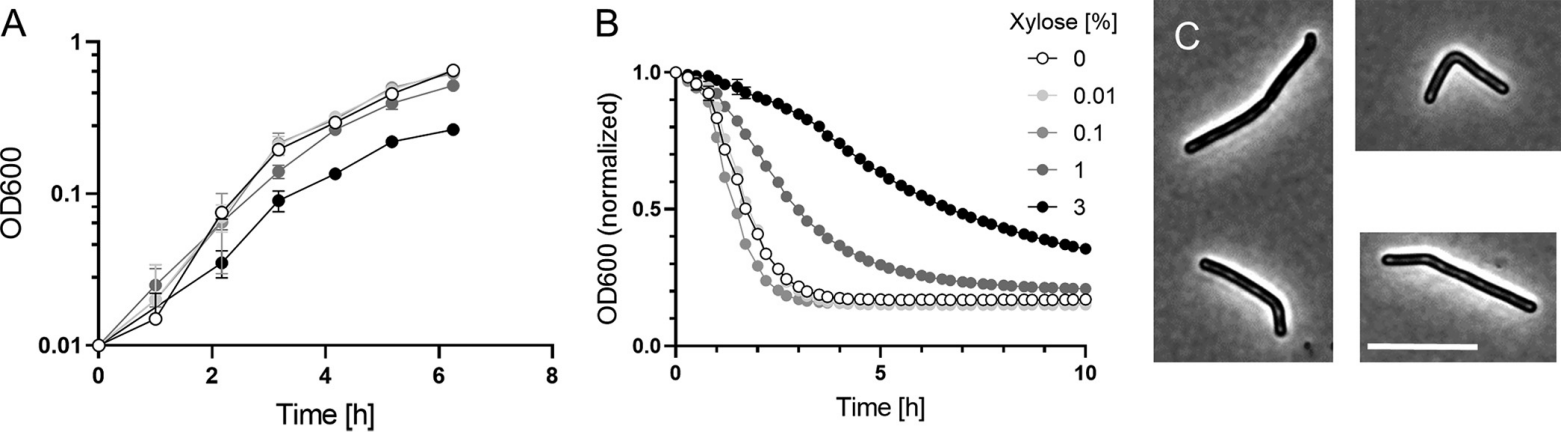
Prolonged overexpression of *walR* from a single-copy chromosomal P*_xyl_*::*walR* allele affects cell shape and slows lysis. (A) Growth curves. An overnight culture of strain UM626 (P*_xyl_*::*walR* integrated at *pyrE* in 630Δ*erm*) was subcultured 1:50 into TY with varied xylose concentrations as indicated, grown for 6 h, and then analyzed in a lysis assay (B) or by phase-contrast microscopy (C). For the lysis assay, cells were suspended in buffer containing 0.01% Triton X-100 and OD_600_ was monitored over time. Data are graphed as the mean and SD of 3 technical replicates, but most error bars are smaller than the symbols. Cells from cultures grown in the presence of 0 to 1% xylose appeared normal and are not shown, but in cultures induced with 3% xylose, about 10% of the cells had irregular or bent morphologies, examples of which are shown in panel C. Bar = 10 μm. Data shown are representative of those from at least 3 independent experiments.

We repeated these experiments after integrating P*_xyl_*::*walR* into the chromosome of 630Δ*erm*. As expected, the effects of xylose induction were similar but less pronounced when P*_xyl_*::*walR* was in single copy (Fig. 4A). The addition of xylose at subculture was now tolerated and reduced growth in a dose-dependent manner. At 3% xylose, cells became slightly phase bright. There were some bent or hooked cells, a defect not observed with the P*_xyl_*::*walR* plasmid, perhaps because it could only be induced for shorter times (Fig. 4C). After 6 h of induction, cells were harvested and evaluated in the lysis assay. We found that overexpression of *walR* slowed lysis in a dose-dependent manner (Fig. 4B).

### Expression profiling under Wal-ON conditions.

As a step toward understanding the basis of the various Wal-related phenotypes, we sought to identify the genes in the *wal* regulon. (Note that we use the term regulon for convenience to refer to genes whose expression responds to manipulation of the *wal* operon regardless of whether these effects are direct or indirect.) We began by performing RNA-seq on two strains that overexpress *walR* from a single-copy P*_xyl_*::*walR* construct integrated at *pyrE*. One of these strains overproduced wild-type WalR; the other overproduced a WalR^D54E^ mutant protein expected to mimic phosphorylated WalR and thus be constitutively active (13, 33) (confusingly, D54 is annotated as D66 in the R20291 genome on the BioCyc website). The two strains behaved similarly with respect to growth and performance in the lysis assay (Fig. S4A and B). The control strain for these experiments had the gene for a red fluorescent protein (*rfp*) integrated at *pyrE* (26). Strains were grown overnight in TY and then subcultured into fresh TY at a starting OD_600_ of ~0.05. When the OD_600_ reached ~0.2, cultures were induced with 3% xylose and allowed to grow for 3 h (~2 mass doublings), at which time cells were harvested and processed for RNA-seq. At this time point, induction of *walR* had only a small effect on growth rate (Fig. S4A) and >99% were viable as judged by LIVE/DEAD staining.

Not including *walR*, which was induced directly from P*_xyl_*, the RNA-seq analysis identified 77 genes whose transcript abundance changed ≥4-fold (*P* < 0.05) upon overexpression of either *walR* or *walR*^D54E^ (Table 2). Of these genes, 44 were induced and 33 were repressed. Induction ratios were very similar in the *walR* and *walR^D54E^* strains with only two exceptions, *cd630_03901* and *cd630_10631*, both of which encode hypothetical proteins. The failure of the D54E substitution to increase gene expression could mean that it does not activate in C. difficile WalR, as reported previously for some other response regulators (34). Alternatively, overexpression of *walR* is itself activating and might obscure the effects of the substitution. The 77 genes are predicted by BioCyc (35) to be organized into 65 operons that contain 18 additional genes which missed our 4-fold expression change cutoff. Visual inspection of the expression data revealed that these genes almost always trended in the right direction, confirming the predicted structure of most of the operons. We therefore included these 18 genes in what we refer to as the Wal-ON regulon, bringing it to a total of 95 genes in 65 operons (Table 2).

**TABLE 2:** Genes affected by Wal-ON conditions

Gene, changed ≥4-fold	Operon^*a*^	Gene name	Annotation^*b*^	Log_2_ fold change			Cell wall association^*d*^
				WalR overexpression		CRISPRi against *wal*^*c*^	
				WalR	WalR^D54E^		
*cd630_07390*	07390		Hypothetical protein	5.26	5.70	−1.38	SPI
*cd630_15220*	15220	*pgdA*	Peptidoglycan deacetylase	4.02	4.26	−1.09	SPI
*cd630_05490*	05490		Hypothetical protein	3.87	4.51	−1.84	SPI
	17810	*walA*	Lipoprotein	NC^*e*^	NC	−2.76	SPII
*cd630_17820*	17820	*walR*	TC response regulator	3.81	3.67	−2.51	
	17830	*walK*	TC sensor histidine kinase	NC	NC	−2.33	Membrane
	17840	*truA2*	tRNA pseudouridine synthase A	NC	NC	−2.34	
*cd630_07380*	07380		Hypothetical protein	3.43	4.14	−1.22	SPI
*cd630_08670*	08670		Hypothetical protein	3.28	3.97	NC	Membrane
*cd630_07400*	07400		PLP-dependent aminotransferase	3.21	2.38	NC	
*cd630_10880*	10880		Hypothetical protein	2.88	3.14	−1.40	Membrane
*cd630_01660*	01660		Peptidase	2.76	2.95	NC	
	01650		Amino acid transporter	NC	NC	NC	Membrane
*cd630_27940*	27940	*cwp12*	Cell wall binding protein	2.69	2.82	NC	SPI
*cd630_33090*	33090	*ligA*	DNA ligase	2.64	2.62	NC	
*cd630_18250*	18250	*metY*	*O*-Acetylhomoserine sulfhydrylase	2.50	2.80	−0.57	
*cd630_18260*	18260	*metA*	Homoserine *O*-succinyltransferase	2.60	2.85	−0.88	
*cd630_14281*	14281		Hypothetical protein	2.59	3.22	NC	
*cd630_27860*	27860	*cwp5*	Cell wall binding protein	2.51	2.65	NC	SPI
*cd630_25380*	25380		Lipoprotein	2.47	2.70	−0.70	SPII
*cd630_03910*	03910	*asnB*	Asparagine synthetase	2.47	3.30	NC	Membrane
*cd630_14960*	14960		Hypothetical protein	2.37	2.86	−0.74	SPI
*cd630_25370*	25370		5′-Nucleotidase/phosphoesterase	2.33	2.49	−0.59	SPI
*cd630_27910*	27910	*cwp2*	Cell wall binding protein	2.30	2.36	−0.59	SPI
	27900		LmbE-like deacetylase	1.56	1.46	−0.55	
	27890	*cwp66*	Cell wall binding protein	1.41	1.42	−0.58	SPI
*cd630_28620*	28620		Peptidase	2.29	2.24	−2.53	
*cd630_34880*	34880	*gtaB1*	UTP-G1P uridylyltransferase	2.23	2.34	−0.73	
*cd630_20580*	20580		Hypothetical protein	2.22	2.43	−1.26	Membrane
*cd630_07410*	07410	*glpK1*	Glycerol kinase	2.21	0.63	NC	
	07420	*eutH*	Ethanolamine utilization protein	1.46	NC	NC	Membrane
	10350	*cwp16*	Amidase, cell wall binding protein	1.24	1.33	NC	SPI
*cd630_10360*	10360	*cwp17*	Amidase, cell wall binding protein	2.15	2.35	NC	SPI
*cd630_21170*	21170	*trxB2*	Thioredoxin reductase	2.12	2.18	NC	
*cd630_00210*	00210	*pycA*	Pyruvate carboxylase	2.10	2.16	−0.46	
*cd630_13890*	13890		Chloromuconate cycloisomerase	2.09	2.18	−0.28	
	13900		Hypothetical protein	1.30	1.64		Membrane
*cd630_22490*	22490		ATPase	2.07	2.20	NC	
*cd630_22480*	22480		Hypothetical protein	1.81	2.04	NC	
*cd630_10890*	10890 10900	*rgbR* *rgbS*	TC response regulator	2.04	2.24	−1.13	
	10900	*rgbS*	TC sensor HK (fragment)	NC	1.16	NC	Membrane
*cd630_30930*	30930		Aminobutyrate hydrolase	2.15	2.35	NC	
	30920		Putative amino acid transporter	1.52	1.02	NC	Membrane
*cd630_26810*	26810		Putative Ca-chelating protein	2.01	2.59	NC	SPI
*cd630_36010*	36010		d-Alanyl–d-alanine carboxypeptidase	1.99	2.35	−1.38	SPI
*cd630_00220*	00220		Elongation factor G	1.97	2.09	NC	
*cd630_14290*	14290		Acyl-CoA N-acyltransferase	1.92	2.47	−1.15	
*cd630_22280*	22280		Hypothetical protein	1.83	2.05	−0.70	Membrane
*cd630_01100*	01100		Hypothetical protein	1.81	2.02	NC	Membrane
	01110		Hypothetical protein	1.51	1.73	NC	SPI
	01111		Hypothetical protein	NC	1.14	NC	
*cd630_09940*	09940		Serine-pyruvate aminotransferase	1.77	2.10	NC	
*cd630_19942*	19942		Hypothetical protein	1.74	2.12	NC	
*cd630_05270*	05270		Beta-lactamase-like hydrolase	1.73	2.13	−0.85	
*cd630_22270*	22270		Radical SAM protein	1.67	2.02	−0.55	SPII
*cd630_18070*	18070		NADPH-dependent FMN reductase	1.49	2.07	−1.53	Membrane
*cd630_03901*	03901		Hypothetical protein	0.92	2.37	NC	
*cd630_10631*	10631		Hypothetical protein	−0.07	2.18	−0.62	Membrane
*cd630_30360*	30360		Major facilitator superfamily transporter	−1.59	−2.13	1.11	Membrane
*cd630_10540*	10540	*bcd2*	Acyl-CoA dehydrogenase	−1.93	−2.23	1.03	
*cd630_10550*	10550	*etfB*	Electron transfer flavoprotein beta	−1.78	−2.11	1.07	
*cd630_10560*	10560	*etfA*	Electron transfer flavoprotein alpha	−1.95	−2.18	1.10	
*cd630_10570*	10570	*crt2*	3-Hydroxybutyryl-CoA dehydratase	−1.96	−2.27	1.06	
*cd630_31000*	31000		C4-dicarboxylate anaerobic carrier	−1.88	−2.59	NC	Membrane
*cd630_30990*	30990		Amidohydrolase	−1.80	−2.47	NC	Membrane
*cd630_10580*	10580	*hbd*	3-Hydroxybutyryl-CoA dehydrogenase	−1.86	−2.05	0.89	SPII
*cd630_10590*	10590	*thlA1*	Acetyl-CoA acetyltransferase	−1.96	−2.16	0.77	
	16820	*queK*	Queuosine hydrolase monomer	−1.31	−1.60	NC	
	16830		ECF transporter S-comp.	−1.39	−1.65	NC	Membrane
*cd630_16840*	16840	*queL*	Radical SAM superfamily protein	−1.90	−2.06	NC	
	23900		BlaI-like	−1.47	−1.65	NC	
*cd630_23890*	23890		BlaR-like	−1.94	−2.26	0.80	Membrane
	23880		Putative PG hydrolase	−1.50	−1.77	NC	SPI
	36040		Hypothetical protein	−0.86	−0.94	0.71	
*cd630_36030*	36030		Phosphoesterase	−1.97	−2.01	1.09	Membrane
*cd630_14040*	14040		Oligopeptide transporter	−2.02	−2.03	NC	Membrane
	14041		Hypothetical protein	−2.02	−1.88	NC	
	25120		PTS, subunit IIA	−1.11	−1.47	NC	
	25110		PTS, antiterminator	−1.45	−1.46	NC	
*cd630_25100*	25100		PTS, subunit IIBC	−2.04	−2.19	0.97	Membrane
*cd630_25320*	25320		Alanine-glyoxylate transaminase	−2.05	−2.34	1.41	
*cd630_25310*	25310		Hypothetical protein	−2.67	−2.75	1.35	Membrane
*cd630_27680*	27680		Cell wall hydrolase	−2.06	−2.29	NC	SPI
*cd630_29660*	29660	*adhE1*	Acetaldehyde-CoA/alcohol dehydrogenase	−2.08	−2.37	−1.09	Membrane
*cd630_18120*	18120		Response regulator	−2.08	−2.28	NC	
*cd630_22600*	22600		Major facilitator superfamily transporter	−2.13	−2.32	NC	Membrane
*cd630_20160*	20160		Hypothetical protein	−2.19	−2.29	4.18	
*cd630_25090*	25090		Glycoside hydrolase	−2.22	−2.33	1.16	
*cd630_21070*	21070		Permease family protein	−2.23	−2.32	NC	Membrane
*cd630_10220*	10220		YczE-like membrane protein	−2.26	−2.73	0.93	Membrane
	10230		Transcriptional regulator	−1.70	−1.94	1.63	
*cd630_10240*	10240	*potA*	ABC transport ATP-binding protein	−2.27	−2.36	1.77	
*cd630_10250*	10250	*potB*	ABC transport permease	−2.47	−2.82	1.72	Membrane
*cd630_10260*	10260	*potC*	ABC transport permease	−2.53	−2.39	1.60	Membrane
*cd630_10270*	10270	*potD*	ABC transport substrate binding protein	−2.29	−2.37	1.55	SPII
*cd630_26640*	26640	*murE*	UDP-*N*-acetylmuramoylalanyl-d-glutamate-2,6-diaminopimelate ligase	−2.85	−3.07	0.93	
*cd630_26670*	26670	*ptsG-BC*	PTS, subunit IIBC	−3.17	−3.52	NC	SPI
*cd630_26660*	26660	*ptsG-A*	PTS, subunit IIA	−3.12	−3.51	NC	
*cd630_21510*	21510		Hypothetical protein	−3.56	−3.82	0.68	Membrane

a Lists other operon members in order.

b TC, two component; PTS, phosphoenolpyruvate-dependent sugar phosphotransferase system; CoA, coenzyme A.

c CRISPRi against *wal*: fold change comparing sgRNA-neg to sgRNA-*walA1* strain in the absence of xylose.

d Reports predictions by SignalP (SPI, predicted to be a substrate of signal peptidase I; SPII, predicted to be a substrate of signal peptidase II) and by BUSCA (membrane). The BUSCA prediction is given only for those genes that were not already predicted to be SPI or SPII substrates by SignalP.

e NC, no change, i.e., the fold change was close to 1 and the *P* value was >0.05.

Several of the genes and trends in Table 2 are worth highlighting. Xylose induction resulted in a 14-fold increase in *walR* mRNA (13-fold for *walR*^D54E^), while the levels of *walK* and other members of the native operon were not affected. This means that induction of P*_xyl_*::*walR* integrated at *pyrE* does not lead to induction of the native *wal* operon, consistent with reports that the *wal* operon is not autoregulated in other bacteria (8, 16). Another striking feature of Table 2 is the abundance of genes encoding cell envelope proteins. Of the 95 genes in Table 2, 22 (23%) are predicted to encode exported proteins and 29 (31%) are predicted to encode cytoplasmic membrane proteins. For comparison, ~7% of the entire proteome is predicted to be exported and 23% predicted to be membrane proteins (see Materials and Methods). Table 2 also includes 21 hypothetical proteins (22% of the total), many of which are likely to be involved in cell envelope processes based on the presence of predicted transmembrane domains or export signals.

Peptidoglycan-associated genes, especially genes encoding PG hydrolases, are prominent members of the Wal regulon in all *Firmicutes* examined to date (8, 10), and C. difficile is no exception. Table 2 lists 10 peptidoglycan-associated genes, including the second most highly induced gene, *pgdA*, which was upregulated 16-fold. PgdA deacetylates *N*-acetylglucosamine in peptidoglycan and is important for C. difficile*’s* high lysozyme resistance (36, 37). AsnB (~8-fold induced) is annotated as an asparagine synthetase, but its primary function is to amidate diaminopimelic acid (*m*-DAP) in PG stem peptides (38). In B. subtilis, amidation of *m*-DAP inhibits PG hydrolase activity (39). MurE (7-fold repressed) catalyzes one of the cytoplasmic steps in PG synthesis, addition of diaminopimelic acid to the nucleotide-linked PG precursor (40). Downregulation of *murE* suggests that PG synthesis may be decreased under Wal-ON conditions. Cwp16 and Cwp17 are S-layer proteins with predicted amidase domains (41) and were upregulated about 4-fold. Cell wall amidases cleave the amide bond that links stem peptides to *N*-acetylglucosamine in PG glycan strands (42). CD630_36010 (4-fold induced) is a predicted d-alanyl–d-alanine carboxypeptidase that removes the terminal d-Ala moiety from peptidoglycan pentapeptide side chains. This might limit 4-3 cross-linking of stem peptides but could favor 3-3 cross-linking. Two proteins of unknown function were upregulated >10-fold, CD630_07390 and CD630_07380. We suspect that both of these proteins are involved in PG metabolism because they are predicted to be exported and also induced by lysozyme (43). Notable among the repressed genes in Wal-ON cells is *cd630_27680*, which is downregulated about 4-fold and is predicted to encode a cell wall hydrolase from the NlpC/P60 family, a domain common to endopeptidases (42). Interestingly, this gene is preceded by two putative WalR binding sites (Table S4), and it has also been characterized as a sortase substrate (44). Another predicted PG hydrolase, CD630_23880, is mildly repressed (~3-fold). Interestingly, the gene encoding this hydrolase is cotranscribed with an uncharacterized BlaI-BlaR regulatory system, *cd630_23890* and *cd630_23900*. Some BlaIR regulatory systems respond to beta-lactam stress (45, 46).

One of the questions that motivated this study was whether the WalR system regulates genes for S-layer proteins in C. difficile. Indeed, the Wal-ON regulon includes six S-layer protein genes, all of which are upregulated by WalR: *cwp2*, -*5*, -*12*, -*16*, -*17*, and -*66*. Two of these genes were mentioned already, the cell wall amidase genes *cwp16* and *cwp17*. The remaining four are of unknown function, although *cwp2* and *cwp12* have domains implicated in adhesion or pathogenesis and studies with *cwp66* have linked it to adhesion, autolysis, and resistance to antibiotics (41, 47). WalRK has been suggested to regulate an S-layer gene in Bacillus anthracis (48).

### Expression profiling under Wal-OFF conditions.

As a complementary approach to identify genes in the WalR regulon, we assessed the effect of CRISPRi knockdown of the *wal* operon on global gene expression. To this end, we performed RNA-seq on a 630Δ*erm* derivative that has the CRISPRi machinery (P*_xyl_*::*dCas9* and P*_gdh_*::*sgRNA-walA1*) integrated into the chromosome at *pyrE*. The control strain was identical except that it expressed an innocuous sgRNA that does not target anywhere in the C. difficile genome (P*_gdh_*::*sgRNA-*neg). Two induction conditions were used. One of these, called “no xylose,” relied on leaky expression of *dCas9*. For this, overnight cultures were diluted into fresh TY to a starting OD_600_ of 0.03 and harvested when they reached an OD_600_ of ~0.7 (Fig. S4C). For the other condition, called “low xylose,” cultures in exponential growth were induced with 0.1% xylose at an OD_600_ of 0.1 and incubated for 2.5 h (~2 mass doublings) before harvest. This induction regimen reduced cell density only modestly (Fig. S4D).

There are 49 genes, organized in 27 operons, that shift 4-fold or more under Wal-OFF conditions (Table 3). The 27 operons contain an additional 14 genes that did not meet our 4-fold cutoff, all but 3 of which nevertheless trended in the right direction. The three exceptions include a pseudogene (*cd630_05260*) that was not detected by our RNA-seq analysis tool and two membrane protein genes (*cd630_05290* and *05280*) whose expression was unchanged. Thus, for the purposes of this analysis, CRISPRi knockdown of the *wal* operon changed the expression of 60 genes, of which 44 were induced and 16 were repressed. Curiously, for most of these genes the fold change was larger in the absence of xylose, suggesting that many of the effects on gene expression might be indirect.

**TABLE 3:** Genes affected by Wal-OFF conditions

Gene, changed ≥4-fold	Operon^*a*^	Gene name	Annotation	Log_2_ fold change			Cell wall association^*c*^
				CRISPRi		WalR OE^*b*^	
				No Xyl	Low Xyl		
*cd630_05140*	05140	*cwpV*	Hemagglutinin/adhesin	4.69	2.74	NC^*d*^	SPI
*cd630_20160*	20160		Hypothetical protein	4.18	1.65	−2.19	
*cd630_11700*	11700		Hypothetical protein	3.52	1.83	NC	
*cd630_11710*	11710	*etfB4*	Electron transfer flavoprotein alpha	3.30	1.40	NC	
*cd630_11720*	11720	*etfA4*	Electron transfer flavoprotein alpha	2.58	1.24	NC	
*cd630_11730*	11730		FAD-linked oxidase	2.17	1.08	NC	
*cd630_08320*	08320	*aksA*	*trans*-Homoaconitate synthase	3.11	2.02	NC	
*cd630_08330*	08330	*acnB*	Aconitate hydratase	3.06	2.05	NC	
*cd630_08340*	08340	*icd*	Isocitrate dehydrogenase	3.07	2.16	NC	
*cd630_17290*	17290		Sodium:phosphate symporter	2.76	0.45	−1.46	Membrane
*cd630_13840*	13840		LamB/YcsF family protein	2.17	0.88	NC	
*cd630_13850*	13850		Hypothetical protein	2.36	1.15	NC	Membrane
*cd630_13860*	13860		Allophanate hydrolase subunit 1	2.52	1.28	NC	
*cd630_13870*	13870		Allophanate hydrolase subunit 2	2.60	1.14	NC	
*cd630_24260*	24260	*buk1*	Butyrate kinase	2.45	NC	−1.41	
	24250	*ptb2*	Phosphate butyryltransferase	1.65	NC		
*cd630_24240*	24240		Pyridoxal phosphate-dependent transferase	2.55	NC	−1.15	
	24230		Putative transporter	1.17	NC		Membrane
*cd630_07650*	07650	*srlEa*	PTS, IIB N-terminal component	2.17	NC	NC	
*cd630_07660*	07660	*srlEb*	PTS, IIB C-terminal component	2.29	NC	NC	Membrane
*cd630_07670*	07670	*srlB*	PTS, IIA subunit	2.27	NC	NC	Membrane
*cd630_07680*	07680	*srlD*	Sorbitol-6-phosphate dehydrogenase	2.35	NC	NC	
	00350		Transcriptional regulator	1.46	0.55	−0.86	
*cd630_00360*	00360	*acoA*	Acetoin dehydrogenase E1 alpha	2.20	0.94	NC	
*cd630_00370*	00370	*acoB*	Acetoin dehydrogenase E1 beta	2.29	0.91	NC	
*cd630_00380*	00380	*acoC*	Acetoin dehydrogenase E2	2.20	0.72	NC	
*cd630_00390*	00390	*acoL*	Acetoin dehydrogenase E3	2.04	0.83	−0.57	
*cd630_30010*	30010		Hydrolase/isomerase/hydratase	2.25	NC	NC	
*cd630_23920*	23920		tRNA-binding protein	2.15	1.65	−1.77	
*cd630_30960*	30960	*bglA3*	6-Phospho-beta-glucosidase	2.13	NC	NC	
*cd630_30950*	30950	*bglA2*	6-Phospho-beta-glucosidase	2.07	NC	NC	
*cd630_25150*	25150		Aspartate aminotransferase	2.12	2.38	−1.76	
	24291		Flavo/ferredoxin oxidoreductase delta	1.19	NC	NC	
*cd630_24290*	24290		Flavo/ferredoxin oxidoreductase alpha	2.11	NC	−0.91	
	24280		Flavo/ferredoxin oxidoreductase beta	1.66	NC	NC	
	24270		Flavo/ferredoxin oxidoreductase gamma	1.50	NC	NC	
*cd630_30040*	30040	*kdgT2*	2-Keto-3-deoxygluconate permease	2.08	0.33	0.93	Membrane
*cd630_30020*	30020	*uxaA*	d-Galactate dehydratase/altronate hydrolase	2.06	0.25	NC	
*cd630_32630*	32630	*pstC*	Phosphate ABC transporter, PstC	2.03	NC	NC	Membrane
	32620	*pstA*	Phosphate ABC transporter, PstA	1.03	NC	NC	Membrane
	32610	*pstB*	Phosphate ABC transporter, ATP binding	1.80	NC	NC	
	32600	*phoU*	Phosphate uptake regulator	1.25	NC	NC	
	30980	*bglG1*	PTS, antiterminator	0.95	NC	NC	
*cd630_30970*	30970	*bglF2*	PTS, subunit IIABC	2.01	NC	−0.76	Membrane
*cd630_28540*	28540	*dltD*	d-Alanine transferase	−1.52	−2.41	1.03	Membrane
*cd630_28530*	28530	*dltA*	d-Ala–d-ala carrier protein ligase 1	−1.34	−2.27	0.84	
*cd630_28520*	28520	*dltB*	d-Alanyl transferase	−1.34	−2.23	0.49	Membrane
	28510	*dltC*	d-Ala–d-ala carrier protein ligase 2	−1.27	−1.89	NC	
*cd630_09000*	09000	*opuCA*	ABC transporter ATP-binding protein	−1.69	−2.36	1.46	Membrane
*cd630_09010*	09010	*opuCC*	ABC transporter permease	−1.68	−2.40	1.27	
*cd630_05190*	05190		Hypothetical protein	−2.00	−2.59	0.57	Membrane
	05260		Aminobenzoyl-glutamate transporter	ND^*e*^	ND	ND	
*cd630_05250*	05250		Peptidase	−2.15	−1.08	NC	
*cd630_17671* *cd630_17680*	17671 17680		Hypothetical protein Membrane protein	−2.18 −2.23	−1.99 −2.18	NC 0.56	Membrane
*cd630_17810*	17810	*walA*	Lipoprotein	−2.76	−5.14	NC	SPII
*cd630_17820*	17820	*walR*	TC response regulator	−2.51	−3.91	3.81	
*cd630_17830*	17830	*walK*	TC sensor histidine kinase	−2.33	−4.03	NC	Membrane
*cd630_17840*	17840	*truA2*	tRNA pseudouridine synthase A	−2.34	−3.92	NC	
*cd630_05300*	05300		Membrane protein	−2.47	NC	NC	Membrane
	05290		Membrane protein	NC	NC	NC	Membrane
	05280		Amidohydrolase	NC	NC	0.92	
*cd630_28620*	28620		Peptidase	−2.53	−1.43	2.29	

a Lists other operon members in order.

b Fold change comparing WalR overexpression to negative-control strain.

c Predictions by SignalP (SPI, predicted to be a substrate of signal peptidase I; SPII, predicted to be a substrate of signal peptidase II) and by BUSCA (membrane). The BUSCA prediction is given only for those genes that were not already predicted to be SPI or SPII substrates by SignalP.

d NC, no change, i.e., the fold change was close to 1 and the *P* value was >0.05.

e ND, CD630_05260 is a pseudogene that was not detected by the RNA-seq analysis tool.

The CRISPRi guide targets the first gene in the operon, *walA*, which was repressed 6.8-fold in the absence of xylose and 35-fold with low xylose. As expected, polarity resulted in repression of the downstream genes as well, ~5-fold with leaky CRISPRi, increasing to ~15-fold with low xylose. The observed polarity confirms the predicted operon structure, including that a seemingly unrelated (and, according to Tn-seq, nonessential) gene for a tRNA modification enzyme is indeed part of the operon.

Unexpectedly, the Wal-OFF gene set comprises mostly transporters and genes for various metabolic functions. Not counting the members of the *wal* operon, only 1 of the genes is predicted to be exported and 15 genes (25%) are expected to be membrane protein genes, a percentage comparable to the genome as a whole (see Materials and Methods). Remarkably, the Wal-OFF regulon does not include a single annotated PG metabolism gene. However, we observed ~5-fold repression of the *dltDABC* operon, which is responsible for the addition of d-alanine to teichoic acids (49). We identified a predicted WalR binding site immediately upstream of *dltDABC*, suggesting direct regulation by WalR. This putative WalR binding site had previously been recognized as one of two direct repeat sequences in the *dlt* promoter region (49) (Table S4). d-Alanylation of teichoic acids affects PG hydrolase activity (50, 51) and vancomycin resistance (52, 53) in some bacteria. In C. difficile, a *dltD* insertion mutant is sensitized to vancomycin and some antimicrobial peptides, but the effects are <2-fold (52). Only one gene in Table 3 codes for an S-layer protein—the hemagglutinin/adhesin gene *cwpV*, which was ~16-fold induced in no xylose. But this gene is subject to phase variation (54), leading us to suspect that it may not be a true member of the WalR regulon.

### Comparison of the Wal-ON and the Wal-OFF gene sets reveals little overlap but opposing trends.

**FIG 5:**
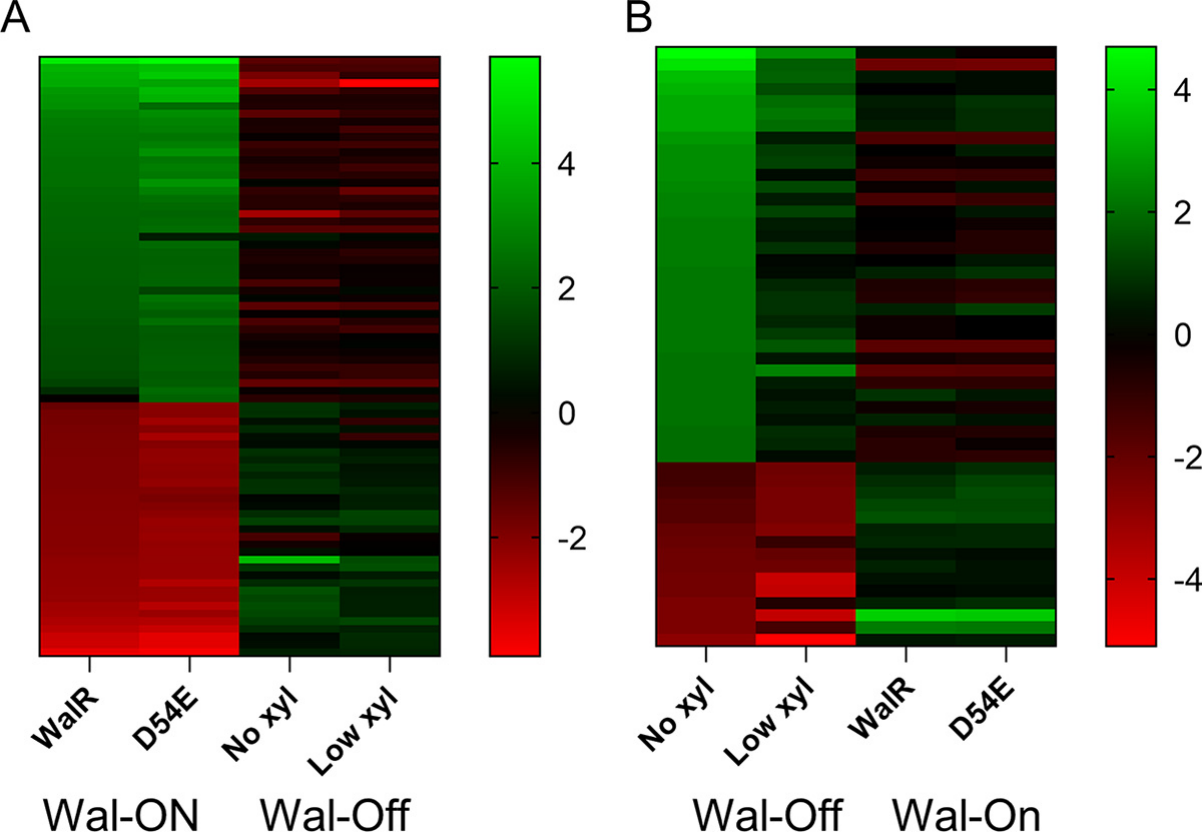
Comparison of transcript changes between Wal-ON and Wal-OFF conditions. (A) Heat map showing the 79 genes whose transcript abundance changed ≥4-fold when *walR* was overexpressed (Wal-ON) in comparison to when the *wal* operon was silenced with CRISPRi (Wal-OFF). (B) Heat map showing the 49 genes whose transcript abundance changed ≥ 4-fold when the *wal* operon was silenced (Wal-OFF) in comparison to when *walR* was overexpressed (Wal-ON). Green, induced; red, repressed.

There are only two proteins common to the Wal-ON and the Wal-OFF gene sets, *cd630_20160* (hypothetical protein) and *cd630_28620* (annotated as a membrane peptidase). However, a large fraction of the 79 Wal-ON genes that met the 4-fold cutoff trended in the opposite direction when evaluated under Wal-OFF conditions, even though the magnitude of change is not as large (Fig. 5A and Table 2). A similar observation was made when genes affected 4-fold or more under Wal-OFF conditions were examined under Wal-ON conditions (Fig. 5B and Table 3).

### Selected members of the WalR regulon were confirmed by plasmid-based reporter fusions.

To confirm results of the RNA-seq analysis, we constructed *rfp* transcriptional fusions (55) to the promoter regions for seven genes from the Wal-ON and Wal-OFF gene sets. Four genes were chosen primarily because they were highly induced when WalR was overexpressed: *cd630_07390*, *pgdA*, *cd630_07380*, and *cd630_08670* (Table 2). Additional considerations included that *pgdA* has an obvious connection to PG biogenesis (36, 37), while *cd630_08670* has a predicted WalR-binding site (Table S4). Two genes were chosen because they were the most strongly repressed upon CRISPRi knockdown of the *wal* operon and are preceded by a predicted WalR-binding site: *cd630_28620* and *cd630_05300* (Table 3 and Table S4). In addition, *cd630_28620* is one of only two genes that made the 4-fold cutoff under both Wal-ON and Wal-OFF conditions. The final gene chosen for confirmation with an *rfp* reporter was *dltD*, which incorporates d-alanine into teichoic acids (49) and was modestly downregulated by CRISPRi knockdown of the *wal* operon. *dltD* stood out because it has a predicted WalR-binding site and is one of the few cell envelope-associated genes that was at least 4-fold repressed under Wal-OFF conditions (Table 3 and Table S4).

Reporter plasmids were constructed by PCR amplifying ~300 bp upstream of the start codon for each selected gene. These fragments contain transcriptional start sites for five of the seven genes (Clost-Base database [56]); start sites for the remaining two genes have not been mapped. The PCR products were cloned into an RFP reporter plasmid. The resulting plasmids were conjugated into the appropriate Wal-ON and Wal-OFF strain pairs: 630Δ*erm* versus 630Δ*erm* P*_xyl_*::*walR* and 630Δ*erm* with sgRNA*-walA1* versus 630Δ*erm* sgRNA-neg. Reporter strains were grown under the same conditions as used for the RNA-seq experiments, except that the Wal-OFF condition was limited to growth in the absence of xylose, i.e., assaying the effect of leaky dCas9 expression on the reporter gene. Harvested cells were fixed and transferred to aerobic conditions to allow RFP to mature. Fluorescence was quantified by flow cytometry.

**FIG 6:**
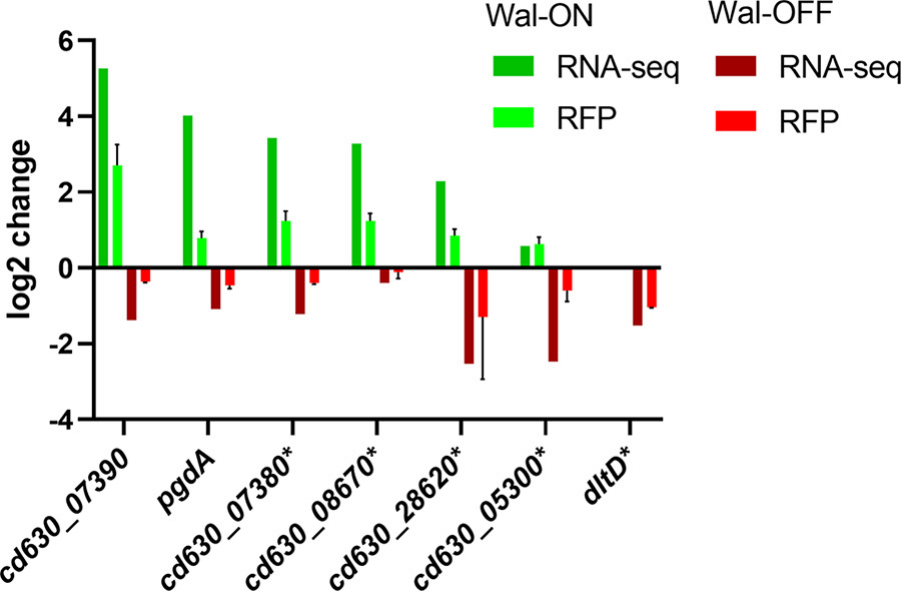
Confirmation of RNA-seq results with RFP reporter fusions to selected genes. Cells harboring plasmids with transcriptional fusions of the red fluorescent protein mCherryOpt to the indicated promoters were grown as per RNA-seq conditions, either Wal-ON (green) or Wal-OFF (red). Red fluorescence was measured by flow cytometry and is graphed as the mean log_2_ fold change from two independent experiments with two technical replicates. Error bars represent the SD of all four measurements. For comparison, the mean log_2_ fold change of the same genes as determined by RNA-seq is also shown. The *dltD* reporter was graphed only under Wal-OFF conditions because this fusion was induced by xylose even in the absence of P*_xyl_*::*walR*. Genes with asterisks represent fusions that were constructed to the corresponding promoter regions from strain R20291.

We found that all RFP reporters responded in the expected direction when *walR* expression was manipulated, thus confirming the RNA-seq results by an orthogonal method (Fig. 6). However, the fold changes were uniformly about 4-fold greater by RNA-seq than by RFP fluorescence. The reasons for this difference are not known. As an aside, having RNA-seq and RFP fluorescence data for the same genes enabled us to show that these are correlated, i.e., baseline RFP fluorescence was higher for genes that returned more reads in RNA-seq experiments (Fig. S5A). This correlation was expected and further indicates that our RNA-seq data are robust.

### Altering the expression level of selected regulon genes does not induce the *wal* reporter.

In B. subtilis, the WalR regulon can be induced or repressed by manipulating expression of *walR*-regulated genes involved in PG metabolism (11, 57). We therefore asked whether manipulating expression of *wal* regulon genes in C. difficile would induce or repress expression of a chromosomal P*_cd630_07390_*::*rfp* reporter. The reporter was chosen because this promoter was highly induced under Wal-ON conditions as determined by RNA-seq (38-fold) or a plasmid-based RFP reporter fusion (7-fold). The chromosomal P*_cd630_07390_*::*rfp* reporter was validated by confirming ~10-fold-increased red fluorescence upon overexpression of *walR* from a P*_xyl_* plasmid (Fig. S5B). Next, we introduced a panel of plasmids that allowed us to directly target 13 WalR regulon genes by overexpression and/or CRISPRi. These 13 genes were chosen for various reasons, including large fold changes in expression, relevance to cell wall biogenesis, and the presence of WalR binding sites (Table S3). Of note, the gene set included genes encoding putative cell wall amidase and the predicted PG hydrolase with an NlpC/P60 domain. Unfortunately, neither overexpression nor CRISPRi knockdown of the selected *wal* regulon genes altered expression of the P*_cd630_07390_*::*rfp* reporter. Further work will be needed to determine whether these negative results reflect technical difficulties or more fundamental differences in Wal signaling between B. subtilis and C. difficile.

### Perturbation of selected Wal regulon genes individually does not lead to any Wal-associated phenotypic defects.

**FIG 7:**
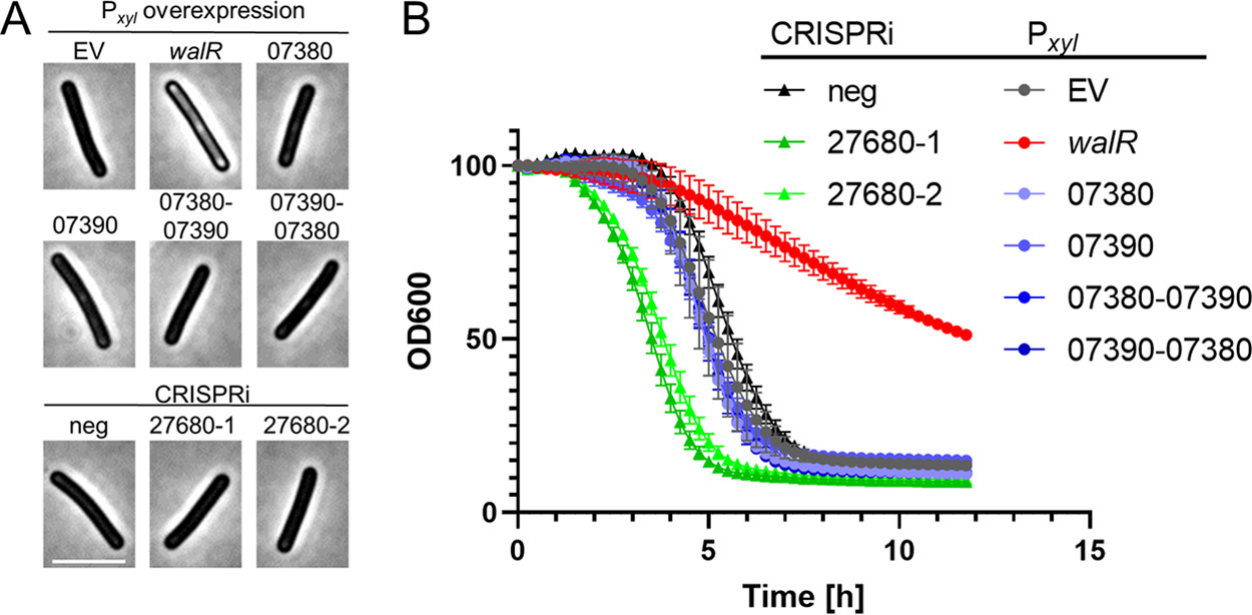
Perturbation of individual Wal-ON regulon genes does not replicate the Wal-ON phenotype. Overnight cultures of 630Δ*erm* harboring overexpression plasmids were subcultured into TY-Thi to an OD_600_ of 0.03, grown to an OD_600_ of 0.2, and induced with 3% xylose for 3 h. Overnight cultures of 630Δ*erm* harboring CRISPRi plasmids were subcultured into TY-Thi containing 1% xylose to an OD_600_ of 0.03 and grown to an OD_600_ of ~0.8. Each culture was then examined by microscopy (bar = 5 μm) (A) and tested in the lysis assay (B). Induction of *walR* is the only condition that achieved phase-bright cells and slowed lysis. The plasmids used were pBZ101 (EV), pCE691 (P*_xyl_*::*walR*), pIA112 (P*_xyl_*::*cd630_07380*), pIA113 (P*_xyl_*::*cd630_07390*), pIA114 (P*_xyl_*::*cd630_07380-07390*), pIA115 (P*_xy_*_l_::*cd630_07390-07380*), pIA34 (sgRNA-neg), pCE744 (*sgRNA-cd630_27680-1*), and pCE745 (*sgRNA-cd630_27680-2*).

As noted above, one motivation for determining the Wal regulon was to identify genes that contribute to Wal phenotypes. Recall that induction of P*_xyl_*::*walR* decreases viability, causes cells to become phase bright, and reduces autolysis in buffer containing 0.01% Triton X-100 (Fig. 3). Two of the genes most highly induced under Wal-ON conditions encode the hypothetical proteins CD630_07380 and CD630_07390. However, overproduction of these proteins either individually or from P*_xyl_* plasmids did not result in any obvious phenotypic changes (Fig. 7). Because these genes are transcribed divergently (35), we needed to reverse the direction of one gene to express them together and decided to clone both possible arrangements: *cd630_07380-cd630_07390* and *cd630_07390-cd630_07380*. Likewise, a CRISPRi plasmid with an sgRNA that targets *cd630_27680* had no effects on growth, morphology, or lysis (Fig. 7); this gene encodes PG hydrolase with two predicted WalR-binding sites and is among the most strongly repressed genes under Wal-ON conditions.

**FIG 8:**
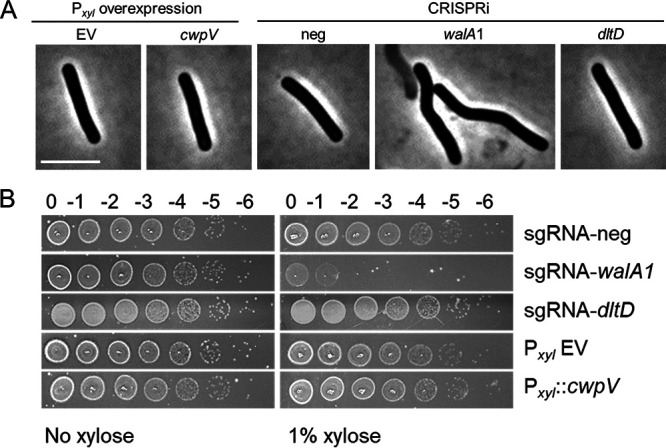
Perturbation of individual Wal-OFF regulon genes does not replicate the Wal-OFF phenotype. (A) Phase-contrast microscopy of strains harboring overexpression plasmids or CRISPRi plasmids after induction with xylose as described in the legend to Fig. 7. Bar = 5 μm. (B) Viability assay. Overnight cultures were serially diluted and spotted onto TY plates with or without 1% xylose. Plates were photographed after incubation overnight. CRISPRi knockdown of the *wal* operon was the only condition that resulted in curved cells, lysis, or a viability defect. The plasmids used were pBZ101 (EV), pCE791 (P*_xyl_*::*cwpV*), pIA34 (sgRNA-neg), pIA50 (sgRNA-*walA1*), and pCE738 (sgRNA-*dltD*).

CRISPRi silencing of the *wal* operon is lethal, and the terminal phenotypes include lysis and loss of rod shape as reflected by an abundance of curved cells (Fig. 2). However, none of these phenotypes were observed when we used a P*_xyl_*::*cwpV* plasmid to overexpress the most highly induced gene from the Wal-OFF RNA-seq data set, which we tested even though *cwpV* is subject to phase variation and might not be part of the WalR regulon. We also observed no effect when we used CRISPRi to knock down expression of *dltD*, which was among the more strongly repressed Wal-OFF genes and is preceded by a putative WalR-binding site (Fig. 8). While this study was not an exhaustive evaluation of all regulon members, our findings suggest that the phenotypic defects observed upon perturbation of the Wal system are not due to changes in expression of any single gene but are cumulative in nature, much like what has been observed in B. subtilis and S. aureus (8–10, 12).

## DISCUSSION

In C. difficile the WalRK TCS is predicted to reside in a four-gene operon: *walA-walR-walK-truA2*. We altered expression of the WalRK regulon by complementary approaches: depleting C. difficile of WalRK by CRISPRi knockdown of the entire operon and overproduction of WalR under P*_xyl_* control. RNA-seq revealed that the *wal* operon is not autoregulated in C. difficile, as evidenced by a lack of induction when WalR (or WalR^D54E^) was ectopically expressed from a P*_xyl_* promoter. Wal operons are not autoregulated in other *Firmicutes* either. RNA-seq also confirmed that the genes constitute an operon, because CRISPRi targeting *walA* resulted in roughly equivalent knockdown of the three downstream genes. Further studies will be needed to establish the specific role of each gene in WalRK signaling. We suspect that *walA* is a bona fide part of the Wal system because all *wal* operons studied to date include accessory genes for membrane proteins that modulate WalK signaling (8, 10). But a rationale for why *truA2*, which encodes a nonessential tRNA modification enzyme, should have come to reside in the *wal* operon is not obvious. This might be the result of evolutionary happenstance.

As expected, extensive knockdown of the *wal* operon with CRISPRi and strong overproduction of WalR from a P*_xyl_* plasmid were both lethal. Phenotypic defects depended on how *walRK* expression was manipulated, but they included elongation, loss of rod shape (curved or wavy cells), phase-bright cells, enhanced or reduced autolysis, and altered sensitivity to antibiotics that target PG synthesis. Similar phenotypes have been observed upon manipulating Wal signaling in other organisms (2, 6, 7, 9, 10, 12, 17, 18). But there are some intriguing differences. In B. subtilis, in which phosphorylated WalR promotes elongation, artificial upregulation of the Wal system causes cells to become elongated, while artificial downregulation causes cells to become short (10, 18). In C. difficile, however, overexpression of WalR from P*_xyl_* had no obvious effect on cell length, while CRISPRi knockdown of the *wal* operon led to elongation.

Vancomycin resistance presents another example of an inverse phenotype. In S. aureus, downregulation of *walRK* expression increases resistance to vancomycin while upregulation of *walRK* decreases vancomycin resistance (58, 59). Vancomycin-intermediate S. aureus (VISA) strains, both laboratory derived (60) and isolated in the clinic (61), often have mutations in *walR* or *walK* that downregulate the system (62). In contrast, we found that partial CRISPRi knockdown of the *wal* operon in C. difficile had the opposite effect, namely, a modest increase in sensitivity to vancomycin. Vancomycin is a front-line treatment for C. difficile infections, so our findings suggest that a small molecule that interferes with Wal signaling might enhance the efficacy of vancomycin therapy.

RNA-seq identified over 150 genes whose expression responds to manipulation of Wal signaling in C. difficile. Although this gene set is diverse, there are themes: many cell envelope proteins, many hypothetical proteins, and about 10 proteins with various connections to PG metabolism. There are also 7 genes for S-layer proteins, consistent with the need to coordinate S-layer assembly and remodeling with growth and turnover of the PG sacculus. The PG hydrolase genes are of particular interest because they are prominent members of the WalR regulon in other *Firmicutes* (5, 12–16) and ectopic expression of one or more of the corresponding enzymes can render *walRK* no longer essential regulators (6, 17, 18). In C. difficile, Wal-ON conditions induced two putative cell wall amidases (*cwp16* and *cwp17*) and repressed one potential d,l-endopeptidase (*cd630_27680*). Direct control of *cd630_27680* by WalR is suggested by the presence of two matches to the WalR-binding site consensus sequence about 100 nucleotides upstream of the start codon (Table S4). The importance of these genes for any Wal-related phenotype is unclear, however, because overexpression and CRISPRi knockdown experiments did not result in any phenotypic changes or alter expression of an RFP reporter that is induced under Wal-ON conditions. In addition to the putative hydrolase genes, several genes in the WalR regulon may play a role in controlling PG hydrolase activity. Deacetylation of PG *N*-acetylglucosamine (GlcNAc) (63), *m*-DAP amidation (39), and d-alanylation of teichoic acids (32, 50, 51, 64) have all been shown to affect hydrolase activity in other organisms.

S. pneumoniae is so far the only organism in which a single essential gene, the PG hydrolase gene *pcsB*, has been identified to be responsible for the essentiality of the *wal* system (6, 7). In B. subtilis, essentiality of the Wal system arises from the cumulative impact of aberrant expression of multiple genes involved in turnover of PG (5, 10, 11). Wal essentiality is also polygenic in nature in S. aureus (8). This seems to be the case in C. difficile as well. Although CRISPRi silencing of the *wal* operon leads to cell lysis, the Wal-OFF gene set (Table 3) does not include any genes determined to be essential by transposon mutagenesis (27). In contrast, the Wal-ON regulon (Table 2) includes 3 essential genes. Two of these are induced by WalR, genes for a DNA ligase (*ligA*, *cd630_33090*) and a putative calcium-chelating protein (*cd630_26810*), and one is repressed, the gene for a PG precursor synthesis protein MurE (*cd630_26640*). It is possible that repression of *murE* contributes to death during overexpression of WalR but not during CRISPRi knockdown of the *wal* operon, because *murE* expression did not change under that condition. Overall, our findings suggest that essentiality of WalRK is driven by a deleterious imbalance of multiple processes. (As an aside, it is worth noting that repression of *murE* under Wal-ON conditions is not what one would expect if WalR promotes increased PG synthesis, as in B. subtilis [11, 57].)

Here, we have provided an initial delineation of Wal phenotypes and the WalR regulon in C. difficile. Our findings raise a number of exciting questions. Is the protein WalA involved in WalRK signaling, and if so, how? What signals are sensed by WalK? Of note, owing to differences in cross-linking (23), the PG hydrolysis products implicated in regulating WalK activity in B. subtilis (11) are not very abundant in C. difficile. Moreover, C. difficile WalK is missing an intracellular PAS domain found in most other WalK proteins, suggesting that C. difficile WalK senses different signals and/or transmits those signals differently. Another open question is which WalR regulon genes are controlled directly by WalR. It will also be important to determine the functions of the WalR-regulated genes, especially those encoding the many hypothetical proteins. Answers to these questions are likely to provide novel insights into cell wall biogenesis and might point the way toward improved therapies against this important pathogen.

## MATERIALS AND METHODS

### Strains, media, and growth conditions.

Bacterial strains are listed in Table 4. C. difficile strains used in this study were derived from either 630Δerm or R20291, both of which have been sequenced. C. difficile was routinely grown in tryptone-yeast extract (TY) medium, supplemented as needed with thiamphenicol at 10 μg/mL (TY-Thi). TY medium consisted of 3% tryptone, 2% yeast extract, and 2% agar (for plates). Brain heart infusion (BHI) medium was prepared per the manufacturer’s (Difco) instructions. C. difficile strains were maintained at 37°C in an anaerobic chamber (Coy Laboratory Products) in an atmosphere of 10% H_2_, 5% CO_2_, and 85% N_2_. Escherichia coli strains were grown in LB medium at 37°C with chloramphenicol at 10 μg/mL (Cam10) or ampicillin at 100 μg/mL (Amp100) as needed. LB medium contained 1% tryptone, 0.5% yeast extract, 0.5% NaCl, and 1.5% agar (for plates). OD_600_ measurements were made with the WPA Biowave CO8000 tube reader in the anaerobic chamber.

**TABLE 4:** Strains used in this study

Strain	Genotype and/or description	Source or reference(s)
E. coli		
OmniMAX-2 T1^R^	F′ [*proAB*^+^ *lacI*^q^ *lacZ*ΔM15 Tn*10* (Tet^r^) Δ(*ccdAB*)] *mcrA* Δ(*mrr-hsdRMS-mcrBC*) φ80(*lacZ*)ΔM15 Δ(*lacZYA-argF*) *U169 endA1 recA1 supE44 thi-1 gyrA96 relA1 tonA panD*	Invitrogen
HB101/pRK24	F^−^ *mcrB mrr hsdS20*(r_B_^−^ m_B_^−^) *recA13 leuB6 ara-14 proA2 lacY1 galK2 xyl-5 mtl-1 rpsL20*	66, 67
C. difficile		
R20291	Wild-type C. difficile strain from UK outbreak (ribotype 027)	
630Δ*erm*	Spontaneous erythromycin-sensitive derivative of strain 630 (ribotype 012)	76
CRG1496	630Δ*erm* Δ*pyrE*	29
UM275	630Δ*erm* with P*_veg_*::*rfp* downstream of *pyrE*	26
UM554	630Δ*erm* with *P_xyl_*::*dCas9 P_gdh_*::*sgRNA-*neg downstream of *pyrE*	This study
UM555	630Δ*erm* with *P_xyl_*::*dCas9 P_gdh_*::*sgRNA-walA-1* downstream of *pyrE*	This study
UM556	630Δ*erm* with *P_xyl_*::*dCas9 P_gdh_*::*sgRNA-walA-2* downstream of *pyrE*	This study
UM626	630Δ*erm* with P*_xyl_*::*walR* downstream of *pyrE*	This study
UM628	630Δ*erm* with P*_xyl_*::*walR^D54E^* downstream of *pyrE*	This study
UM926	630Δ*erm* with P*_cd630_0739_*::*mCherryOpt* downstream of *pyrE*	This study

### Plasmid and strain construction.

All plasmids are listed in Table 5; an expanded version of this table which includes additional information relevant to plasmid assembly is provided in Table S2A in the supplemental material. Plasmids were constructed by isothermal assembly (65) using reagents from New England Biolabs (Ipswich, MA). Regions of plasmids constructed using PCR were verified by DNA sequencing. The oligonucleotide primers used in this work were synthesized by Integrated DNA Technologies (Coralville, IA) and are listed in Table S2B. All plasmids were propagated using OmniMax 2-T1R as the cloning host, transformed into HB101/pRK24 (66, 67), and then introduced into C. difficile strains by conjugation. Chromosomal fusions at the *pyrE* locus in 630Δerm were constructed by allelic exchange (29) using C. difficile CRG1496 (630Δ*erm* Δ*pyrE*) as a *pyrE*-deficient recipient. The allelic exchange restored a functional *pyrE* gene.

**TABLE 5:** Plasmids used in this study

Plasmid	Relevant features	Reference or comment
pAP114	P*_xyl_*::*mCherryOpt catP*	26
pBZ101	P*_xyl_* empty vector	This study
pCE636	P*_dltD_*::*mCherryOpt catP*	This study
pCE738	P*_xyl_*::*dCas9-opt* P*_gdh_*::*sgRNA-dltD-1 catP*	37
pCE691	P*_xyl_*::*walR catP*	This study
pCE738	P*_xyl_*::*dCas9-opt* P*_gdh_::sgRNA-dltD-1 catP*	37
pCE741	P*_xyl_*::*cdr2656*	This study
pCE744	P*_xyl_*::*dCas9-opt* P*_gdh_*::*sgRNA-cd630_27680-1 catP*	This study
pCE745	P*_xyl_*::*dCas9-opt* P*_gdh_*::*sgRNA-cd630_27680-2 catP*	This study
pCE789	P*_xyl_*::*dCas9-opt* P*_gdh_*::*cwpV-1 catP*	This study
pCE791	P*_xyl_*::*cwpV*	This study
pDSW1728	P*_tet_*::*mCherryOpt catP*	55
pDSW2037	P*_xyl_* in integration vector pMTL-YN1C *catP*	This study
pDSW2053	P*_xyl_*::*dCas9opt-P_gdh_*::*sgRNA*-neg in pMTL-YN1C *catP*	This study
pDSW2055	P*_xyl_*::*dCas9opt-P_gdh_*::*sgRNA-cd630_17810-1* in pMTL-YN1C *catP*	This study
pDSW2057	P*_xyl_*::*dCas9opt-P_gdh_*::*sgRNA-cd630_17810-2* in pMTL-YN1C *catP*	This study
pIA33	P*_xyl_*::*dCas9-opt* P*_gdh_*::*sgRNA-rfp catP*	26
pIA34	P*_xyl_*::*dCas9-opt* P*_gdh_*::*sgRNA*-neg *catP*	26
pIA50	P*_xyl_*::*dCas9-opt* P*_gdh_*::*sgRNA-walA-1 catP*	This study
pIA51	P*_xyl_*::*dCas9-opt* P*_gdh_*::*sgRNA-walA-2 catP*	This study
pIA75	P*_xyl_*::*walR* in pMTL-YN1C *catP*	This study
pIA76	P*_xyl_*::*walR^D54E^* in pMTL-YN1C *catP*	This study
pIA79	P*_xyl_*::*dCas9-opt* P*_gdh_*::*cd630_25040-1 catP*	This study
pIA80	P*_xyl_*::*dCas9-opt* P*_gdh_*::*cd630_25040-2 catP*	This study
pIA81	P*_xyl_*::*dCas9-opt* P*_gdh_*::*cd630_36010-1 catP*	This study
pIA93	P*_cdr_0665_*::*mCherryOpt catP*	This study; *cdr_0665* is ortholog of *cd630_07380*
pIA95	P*_cdr_0796_*::*mCherryOpt catP*	This study; *cdr_0796* is ortholog of *cd630_08670*
pIA97	P*_cdr_2753_*::*mCherryOpt catP*	This study; *cdr_2753* is ortholog of *cd630_28620*
pIA98	P*_cdr_0455_*::*mCherryOpt catP*	This study; *cdr_0455* is ortholog of *cd630_53000*
pIA100	P*_cd630_0739_*::*mCherryOpt catP*	This study
pIA101	P*_cd630_0739_*::*mCherryOpt* in pMTL-YN1C *catP*	This stduy
pIA102	P*_xyl_*::*dCas9-opt* P*_gdh_*::*cd630_05490 catP*	This study
pIA103	P*_xyl_*::*dCas9-opt* P*_gdh_*::*cd630_27940 catP*	This study
pIA104	P*_xyl_*::*dCas9-opt* P*_gdh_*::*cd630_27860 catP*	This study
pIA105	P*_xyl_*::*dCas9-opt* P*_gdh_*::*cd630_27910 catP*	This study
pIA106	P*_xyl_*::*dCas9-opt* P*_gdh_*::*cd630_10360 catP*	This study
pIA107	P*_pgdA_::mCherryOpt catP*	This study
pIA108	P*_xyl_*::*dCas9-opt* P*_gdh_*::*cd630_07380-1 catP*	This study
pIA109	P*_xyl_*::*dCas9-opt* P*_gdh_*::*cd630_07380-2 catP*	This study
pIA110	P*_xyl_*::*dCas9-opt* P*_gdh_*::*cd630_07390-1 catP*	This study
pIA111	P*_xyl_*::*dCas9-opt* P*_gdh_*::*cd630_07390-2 catP*	This study
pIA112	P*_xyl_*::*cd630_0738*	This study
pIA113	P*_xyl_*::*cd630_0739*	This study
pIA114	P*_xyl_*::*cd630_0738-cd630_0739*	This study
pIA115	P*_xyl_*::*cd630_0739-cd630_0738*	This study
pMTL-YN1C	E. coli*-*C. difficile shuttle vector for inserting genes into C. difficile chromosome while restoring *pyrE*; c*olE1 RP4oriT-TraJ CB102ori-repH′ catP*	29

### Conjugation into C. difficile.

Our experiments required conjugating plasmids into C. difficile strains R20291 and 630Δ*erm*. In the case of R20291, we encountered problems with low conjugation efficiencies even with heat shock as described previously (68). After testing several modifications of the procedure, we found that performing conjugations on filters reliably increased efficiency more than 10-fold (Fig. S6). We now routinely use filters for all our conjugations. Briefly, the E. coli donor strain (an HB101/pRK24 derivative harboring the cargo plasmid) was grown overnight in LB Amp^100^ Cam^10^. The donor strain was collected gently by centrifugation of a 0.5-mL aliquot at 5,000 × *g* for 1 min and then washed with 1 mL of TY and pelleted again. The washed cell pellet was moved into the anaerobic chamber. R20291 was prepared for conjugation by a heat shock step (68). For this, a 200-μL aliquot of the overnight culture of the R20291 recipient was transferred to a 1.5-mL microcentrifuge tube and incubated at 48°C for 5 min in a Fisherbrand dry bath (with water-filled wells). Then 100 μL of heat-shocked R20291 was used to take up the pellet of E. coli donor cells. The strain mixture was then pipetted onto a 25-mm-diameter, 0.45-μm-pore-size Millipore mixed-cellulose filter (HAWP02500) placed on BHI plates. After incubation for 24 h at 37°C, cells were flushed from the membrane with 500 μL of TY and then 200 to 500 μL of the resulting bacterial slurry was plated onto TY amended with thiamphenicol (10 μg/mL), kanamycin (50 μg/mL), and cefoxitin (8 μg/mL) to select for exconjugants. Conjugations into 630Δ*erm* were done identically except that the heat shock step was omitted.

### Viability assay.

The effect of CRISPRi silencing on plating efficiency was evaluated by making a 10-fold serial dilution of a culture grown overnight in TY-Thi and spotting 5 μL of each dilution onto TY-Thi agar with and without 1% xylose. Plates were photographed after overnight incubation (~18 h).

### Microscopy.

Cells were immobilized using thin agarose pads (1%). Phase-contrast and fluorescent micrographs were recorded on an Olympus BX60 microscope equipped with a 100× UPlanApo objective (numerical aperture, 1.35). Micrographs were captured with a Hamamatsu Orca Flash 4.0 V2+ complementary metal oxide semiconductor (CMOS) camera. The image analysis tool MicrobeJ (69) was used to measure cell length and sinuosity, which is the length of the cell axis divided by the distance between the poles. A perfectly straight rod has a sinuosity value of 1, while a curved or wavy cell has a larger value. We classified cells as curved if their sinuosity was ≥1.03 (see Fig. S2 for examples). Viability of cells was assessed with LIVE/DEAD stain (Molecular Probes; L7012). A 1-mL culture sample was pelleted, washed with phosphate-buffered saline (PBS), and resuspended in 100 μL of PBS with 5 μM Syto 9 and 30 μM propidium iodide. Cells were incubated with the dyes for 15 min, then removed from the anaerobic chamber, and immediately imaged by microscopy. For propidium iodide red fluorescence we used filter set 41004 (Chroma Technology) with a 538- to 582-nm excitation filter, 595-nm dichroic mirror (long pass), and a 582- to 682-nm emission filter. Syto 9 green fluorescence was captured with filter set 41017 (Chroma Technology Corp.) with a 450- to 490-nm excitation filter, a 495-nm dichroic mirror (long pass), and a 500- to 550-nm emission filter.

### Fixation protocol.

A 500-μL aliquot of cells in growth medium was added directly to a microcentrifuge tube containing 120 μL of a 5× fixation cocktail: 100 μL of a 16% (wt/vol) paraformaldehyde aqueous solution (Alfa Aesar, Ward Hill, MA) and 20 μL of 1 M NaPO_4_ buffer (pH 7.4). The sample was mixed, incubated in the anaerobic chamber at 37°C for 30 min and then on ice 30 min, and removed from the chamber. The fixed cells were washed twice with 1 mL of PBS, resuspended in 50 μL of PBS, and left in the dark for 18 h to allow for chromophore maturation.

### MIC determination.

Antibiotic sensitivity was determined in 96-well plates. A 2-fold dilution series of selected antibiotics was prepared in 50 μL TY medium. Wells were then inoculated with 50 μL of a diluted culture suspension (10^6^ CFU/mL; calculated OD_600_, ~0.005). Plates were imaged and evaluated after 17 h at 37°C.

### Lysis assay.

Cell cultures (1 mL) were removed from the anaerobic chamber, pelleted, and resuspended in 700 μL of 0.01% Triton X-100 in 50 mM NaPO_4_ buffer (pH 7.4). Of this 700 μL, three 200-μL replicates were pipetted into wells of a clear, flat-bottom 96-well plate. The turbidity was measured at 600 nm every 15 min for 10 h in a plate reader (Tecan Infinite M200 Pro).

### Flow cytometry.

Cells were analyzed at the Flow Cytometry Facility at the University of Iowa using the Becton, Dickinson LSR II instrument with a 561-nm laser, a 610/20-nm-band-pass filter, and a 600 LP dichroic filter. Data were analyzed using BD FACSDiva software.

### Culture growth for RNA-seq samples.

To obtain robust, quality data, all experiments were performed on three different days for biological replicates. Cultures were grown in 100 mL of TY as follows. For the Wal-ON condition, to characterize the effect of overexpressing *walR*, strains were subcultured to an OD_600_ of ~0.05, grown to an OD_600_ of 0.2, induced with xylose to 3%, and grown for an additional 3h (Fig. S4A). For the Wal-OFF condition, to characterize the effect of partial knockdown of the *wal* operon under leaky CRISPRi conditions, strains were subcultured to an OD_600_ of ~0.03 and grown to an OD_600_ of ~0.7 before harvesting (Fig. S4C). To test the effect of stronger knockdown of the *wal* operon, growth conditions were identified that allowed exposing cells to moderate levels of xylose (0.1%) without a significant loss of biomass. Strains were subcultured to an OD_600_ of ~0.03, grown to an OD_600_ of 0.3, diluted back to an OD_600_ of ~0.1, induced with xylose to a final concentration of 0.1%, and harvested after an additional 2.5 h of growth (Fig. S4D). From each condition, 25 mL of culture was fixed by rapidly mixing with 25 mL of ice-cold 1:1 ethanol-acetone that had been brought into the anaerobic chamber on dry ice. Fixed cells were stored at −80°C until further workup.

### RNA isolation and RNA-seq.

Fixed culture samples were thawed and then pelleted for 10 min at 8,000 × *g* and 4°C, and pellets were washed once with 500 μL of 1% β-mercaptoethanol (β-ME) in water. Subsequent steps were an adaptation of the Qiagen RNeasy minikit procedure. The washed pellet was resuspended in 1 mL of the Qiagen-provided RLT buffer amended with 1% β-ME and transferred to a 2-mL screw-cap tube filled with glass beads (0.1-mm diameter) to a height of ~2 to 3 mm. Cells were lysed with the FastPrep-24 homogenizer with two cycles at 6 m/s for 45 s, resting on ice for 3 min between cycles. The homogenized material was pelleted at 14,000 × *g* for 15 min at 4°C. The supernatant was removed, adjusted to 900 μL with RLT buffer/β-ME, mixed with 500 μL of ethanol, and loaded onto the Qiagen RNeasy spin column. Subsequent steps followed the manufacturer’s protocol. The RNA was eluted with 50 μL of water and DNase treated twice (Ambion Turbo DNA free). Absence of DNA was evaluated by amplifying a 364-bp region of *mldA* with *Taq* DNA polymerase for 30 cycles. The RNA integrity number (RIN) was determined with an Agilent Bioanalyzer at the Iowa Institute for Human Genetics, Genomics Division. All samples had a RIN of 9.4 or higher. Typical yields from 25 mL of culture were 8 to 20 μg of RNA at 200 to 500 ng/μL.

RNA samples were submitted to the Microbial Genome Sequencing Center (MiGS) in Pittsburgh, PA. MiGS performed Illumina stranded RNA library preparation paired with RiboZero Plus (per the manufacturer’s specifications) and sequenced on a NextSeq 500 using a 75cyc high-output flow cell. Fastq files were trimmed and filtered using a combination of Trimmomatic (70) and FastQC (https://www.bioinformatics.babraham.ac.uk/projects/fastqc/). Alignment, normalization, and differential expression were analyzed with SeqMan NGen version 17.2 (DNASTAR, Madison, WI). Annotation was imported from NCBI with additional information obtained from progenomes (71). All subsequent analysis was performed in Excel and GraphPad Prism 9.

### Bioinformatics.

Gene sequences and operon organization were obtained from the BioCyc data collection (35, 72). SignalP 5.0 (73) was used to search for type I and type II signal peptides. BUSCA (74) was used to predict protein cellular location. When all C. difficile proteins were run through the prediction programs, SignalP predicted ~7% to be exported. BUSCA predicted ~4% to exported and ~25% to be membrane associated. Cell wall association in Tables 2 and 3 was reported as follows: (i) signal peptidase substrate if predicted by SignalP and (ii) for all remaining genes, membrane protein if predicted by BUSCA.

Putative WalR binding sites were identified with Virtual Footprint (75) by querying with (i) the B. subtilis consensus motif, TGTWAH-N5-TGTWAH (14), (ii) the same motif, but allowing one mismatch, or (iii) TGTNDH-N5-BKBWRN (8). Searches were limited to the intergenic region of the genome and generated 29, 522, and 684 hits, respectively.

### Data availability.

RNA-seq data were submitted to the NCBI GEO repository and assigned accession number GSE200346.
