# Supplementary material for: The WalRK Two-Component System Is Essential for Proper Cell Envelope Biogenesis in Clostridioides difficile
Source: J Bacteriol. 2022 May 16;204(6):e00121-22. doi: 10.1128/jb.00121-22 (PMC9210968; doi:10.1128/jb.00121-22)
Supplement: Supplemental file 2 — Tables S2 to S4, Fig. S1 to S6. Download jb.00121-22-s0002.pdf, PDF file, 0.8 MB [file jb.00121-22-s0002.pdf]

Table S2 Plasmids used in this study. A. Construction details.

Plasmid	Relevant features	Parent vector	Restriction enzymes to digest parent vector	PCR primers	PCR template	Assembly	Comments	Reference
pAP114	P_{xyl} ::*mCherryOpt catP*							(26)
pBZ101	P_{xyl} empty vector	pAP114	BamHI, SacI			Ligation	Blunt end with Klenow, ligate with T4 DNA ligase	
pCE636	P_{dltD} ::*mCherryOpt catP*	pDSW1728	KpnI, SacI	4141+4142	R20291	ITA	191 nucleotides upstream of dltD (cdr_2745) cloned before mCherry reporter	This study
pCE655	P_{xyl} ::*dCas9-opt* P_{gdh} ::*sgRNA-pgdA-1 catP*							(37)
pCE691	P_{xyl} ::*walR catP*	pAP114	SacI, BamHI	4546+4547	R20291	ITA	*walR* from R20291 cloned under xylose control	This study
pCE738	P_{xyl} ::*dCas9-opt* P_{gdh} ::*sgRNA-dltD-1 catP*							(37)
pCE741	P_{xyl} ::*cdr2656*	pAP114	BamHI, SacI	4722+4723	R20291	ITA	cdr_2656 (ortholog of cd630_27680) under xylose control	This study
pCE744	P_{xyl} ::*dCas9-opt* P_{gdh} ::*sgRNA-cd630_27680-1 catP*	pIA33	MscI, NotI	4719+4084	pIA33	ITA	Replace sgRNA-rfp with sgRNA-cd630_27680-1	This study
pCE745	P_{xyl} ::*dCas9-opt* P_{gdh} ::*sgRNA-cd630_27680-2 catP*	pIA33	MscI, NotI	4720+4084	pIA33	ITA	Replace sgRNA-rfp with sgRNA-cd630_27680-2	This study
pCE789	P_{xyl} ::*dCas9-opt* P_{gdh} ::*cwpV-1 catP*	pIA33	MscI, NotI	4939+4084	pIA33	ITA	Replace sgRNA-rfp with sgRNA-cd630_cwpV	This study
pCE791	P_{xyl} ::*cwpV*	pAP114	BamHI, SacI	4937+4938	R20291	ITA	*cwpV* from R20291 under xylose control	This study
pDSW1728	P_{tet} ::*mCherryOpt catP*							(55)
pDSW2037	P_{xyl} in integration vector pMTL-YN1C *catP*	pMTL-YN1c	SacI, SalI	P2405+P2406	R20291	Ligation	Vector suitable for inserting genes under P_{xyl} -control at *pyrE* in 630Δerm	This study
pDSW2053	P_{xyl} ::*dCas9opt-P* $_{gdh}$ ::*sgRNA-neg* in pMTL-YN1C *catP*	pDSW2037	SalI+NheI	P2408+P2411	pIA34	Ligation	Vector suitable for integration at *pyrE* in 630Δerm, cargo: P_{xyl} ::*dCas9opt-* P_{gdh} ::*sgRNA-neg*	This study
pDSW2055	P_{xyl} ::*dCas9opt-P* $_{gdh}$ ::*sgRNA-cd630_17810-1* in pMTL-YN1C *catP*	pDSW2053	SalI+NheI	P2408+P2411	pIA50	Ligation	Vector suitable for integration at *pyrE* in 630Δerm, cargo: P_{xyl} ::*dCas9opt-* P_{gdh} ::*sgRNA-cd630_17810-1*	This study
pDSW2057	P_{xyl} ::*dCas9opt-P* $_{gdh}$ ::*sgRNA-cd630_17810-2* in integration vector pMTL-YN1C *catP*	pDSW2053	SalI+NheI	P2408+P2411	pIA51	Ligation	Vector suitable for integration at *pyrE* in 630Δerm, cargo: P_{xyl} ::*dCas9opt-* P_{gdh} ::*sgRNA-cd630_17810-2*	This study
pIA33	P_{xyl} ::*dCas9-opt* P_{gdh} ::*sgRNA-rfp catP*							(26)
pIA34	P_{xyl} ::*dCas9-opt* P_{gdh} ::*sgRNA-neg catP*							(26)
pIA50	P_{xyl} ::*dCas9-opt* P_{gdh} ::*sgRNA-cd630_17810-1 catP*	pIA33	MscI, NotI	4454+4084	pIA33	ITA	Replace sgRNA-rfp with sgRNA-cd630_17810-1; will also target cdr_1676	This study
pIA51	P_{xyl} ::*dCas9-opt* P_{gdh} ::*sgRNA-cd630_17810-2 catP*	pIA33	MscI, NotI	4455+4084	pIA33	ITA	Replace sgRNA-rfp with sgRNA-cd630_17810-2; will also target cdr_1676	This study
pIA75	P_{xyl} ::*walR* in integration vector pMTL-YN1C *catP*	pDSW2037	HindIII, SalI	4658+4659	pCE691	ITA	Vector suitable for integration at *pyrE* in 630Δerm, cargo: P_{xyl} ::*walR*	This study
pIA76	P_{xyl} :*walR-D66E* in integration vector pMTL-YN1C *catP*	pDSW2037	HindIII, SalI	4658+4660, 4661+4659	pCE691	ITA	Vector suitable for integration at *pyrE* in 630Δerm, cargo: P_{xyl} ::*walR* -D66E	This study
pIA79	P_{xyl} ::*dCas9-opt* P_{gdh} ::*cd630_25040-1 catP*	pIA33	MscI, NotI	5110+4084	pIA33	ITA	Replace sgRNA-rfp with sgRNA-cd630_25040-1	This study
pIA80	P_{xyl} ::*dCas9-opt* P_{gdh} ::*cd630_25040-2 catP*	pIA33	MscI, NotI	5111+4084	pIA33	ITA	Replace sgRNA-rfp with sgRNA-cd630_25040-2	This study
pIA81	P_{xyl} ::*dCas9-opt* P_{gdh} ::*cd630_36010-1 catP*	pIA33	MscI, NotI	5112+4084	pIA33	ITA	Replace sgRNA-rfp with sgRNA-cd630_36010-1	This study

Table S2 Plasmids used in this study. A. Construction details.

Plasmid	Relevant features	Parent vector	Restriction enzymes to digest parent vector	PCR primers	PCR template	Assembly	Comments	Reference
pIA93	P_{cdr_0665} *::mCherryOpt catP*	pDSW1728	KpnI, SacI	5394+5395	R20291	ITA	300 nucleotides upstream of cdr_0665 (ortholog of CD630_07380) plus first two codons cloned before mCherry reporter	This study
pIA95	P_{cdr_0796} *::mCherryOpt catP*	pDSW1728	KpnI, SacI	5375+5376	R20291	ITA	300 nucleotides upstream of cdr_0796 (ortholog of CD630_08670) plus first two codons cloned before mCherry reporter	This study
pIA97	P_{cdr_2753} *::mCherryOpt catP*	pDSW1728	KpnI, SacI	5396+5397	R20291	ITA	300 nucleotides upstream of cdr_2753 (ortholog of CD630_28620) plus first two codons cloned before mCherry reporter	This study
pIA98	P_{cdr_0455} *::mCherryOpt catP*	pDSW1728	KpnI, SacI	5398+5399	R20291	ITA	300 nucleotides upstream of cdr_0455 (ortholog of CD630_53000) plus first two codons cloned before mCherry reporter	This study
pIA100	P_{cd630_0739} *::mCherryOpt catP*	pDSW1728	KpnI, SacI	5420+5421	630Δerm	ITA	300 nucleotides upstream of CD630_07390 plus first two codons cloned before mCherry reporter	This study
pIA101	P_{cd630_0739} *::mCherryOpt* in pMTL-YN1C *catP*	pMTL-YN1C	NotI, HindIII	5485+5486	pIA100	ITA	Vector suitable for integration at *pyrE* in 630Δerm, cargo: P_{cd630_07390} *::mCherryOpt*	This study
pIA102	P_{xyl}*::dCas9-opt* P_{gdh}*::cd630_05490 catP*	pIA33	MscI, NotI	5641+4084	pIA33	ITA	Replace sgRNA-rfp with sgRNA-cd630_05490	This study
pIA103	P_{xyl}*::dCas9-opt* P_{gdh}*::cd630_27940 catP*	pIA33	MscI, NotI	5642+4084	pIA33	ITA	Replace sgRNA-rfp with sgRNA-cd630_27940 (cwp12)	This study
pIA104	P_{xyl}*::dCas9-opt* P_{gdh}*::cd630_27860 catP*	pIA33	MscI, NotI	5643+4084	pIA33	ITA	Replace sgRNA-rfp with sgRNA-cd630_27860 (cwp5)	This study
pIA105	P_{xyl}*::dCas9-opt* P_{gdh}*::cd630_27910 catP*	pIA33	MscI, NotI	5644+4084	pIA33	ITA	Replace sgRNA-rfp with sgRNA-cd630_27910 (cwp2)	This study
pIA106	P_{xyl}*::dCas9-opt* P_{gdh}*::cd630_10360 catP*	pIA33	MscI, NotI	5645+4084	pIA33	ITA	Replace sgRNA-rfp with sgRNA-cd630_10360 (cwp17)	This study
pIA107	P_{pgdA} *::mCherryOpt catP*	pDSW1728	KpnI, SacI	5664+5665	630Δerm	ITA	300 nucleotides upstream of pgdA (CD630_15220) plus first two codons cloned before mCherry reporter	This study
pIA108	P_{xyl}*::dCas9-opt* P_{gdh}*::cd630_07380-1 catP*	pIA33	MscI, NotI	5705+4084	pIA33	ITA	Replace sgRNA-rfp with sgRNA-cd630_380-1	This study
pIA109	P_{xyl}*::dCas9-opt* P_{gdh}*::cd630_07380-2 catP*	pIA33	MscI, NotI	5707+4084	pIA33	ITA	Replace sgRNA-rfp with sgRNA-cd630_380-2	This study
pIA110	P_{xyl}*::dCas9-opt* P_{gdh}*::cd630_07390-1 catP*	pIA33	MscI, NotI	5706+4084	pIA33	ITA	Replace sgRNA-rfp with sgRNA-cd630_390-1	This study
pIA111	P_{xyl}*::dCas9-opt* P_{gdh}*::cd630_07390-2 catP*	pIA33	MscI, NotI	5708+4084	pIA33	ITA	Replace sgRNA-rfp with sgRNA-cd630_390-2	This study
pIA112	P_{xyl} *::cd630_0738*	pAP114	BamHI, SacI	5715+5726	630Δerm	ITA	cd630_0738 from 630Δerm under xylose control	This study
pIA113	P_{xyl} *::cd630_0739*	pAP114	BamHI, SacI	5717+5718	630Δerm	ITA	cd630_0739 from 630Δerm under xylose control	This study
pIA114	P_{xyl} *::cd630_0738-cd630_0739*	pAP114	BamHI, SacI	5715+5719, 5720+5718	630Δerm	ITA	cd630_0738 and cd630_0739 from 630Δerm under xylose control; separate Shine Dalgano sequence	This study
pIA115	P_{xyl} *::cd630_0739-cd630_0738*	pAP114	BamHI, SacI	5717+5721, 5722+5725	630Δerm	ITA	cd630_0739 and cd630_0738 from 630Δerm under xylose control; separate Shine Dalgano sequence	This study
pMTL-YN1C	*E. coli-C. difficile* shuttle vector for inserting genes into *C. difficile* chromosome while restoring *pyrE*; *colE1 RP4oriT-TraJ CB102ori-repH' catP*							(29)

Table S2 B. Oligonucleotides

Oligo	Sequence	Relevant features
CDEP4084	AACTTATAGGATCCGCGGCCGCTAGTCAGACATCATGCTGATCTAGA	Underlined: BamHI site
CDEP4141	GGCTTCTTATTTTTATGGTACAAATTATAAGTATGAAAAAGTGCTAAAAAG	
CDEP4142	TCTCCTTTACTGCAGGAGCTATTTTCTCTCCTCTAAAAATATTCAAAT	
CDEP4454	AATTAAACTGTAAATGGCCA **AGTAACAAATAACATCATCA** GTTTTAGAGCTAGAAATAGC	Underlined: MscI; Bold: sgRNA-cdr_1676-1
CDEP4455	AATTAAACTGTAAATGGCCA **GGCAATATCATAGATGAATC** GTTTTAGAGCTAGAAATAGC	Underlined: MscI; Bold: sgRNA-cdr_1676-2
CDEP4546	CGATAGTTATGAAGTGAGCTTAAGGAGGATTAATATATGGTTAATATTATACTCAATTGGA	
CDEP4547	GTTTTATTAAAACTTATAGGATCCAGACCTTCAAAATGTAACACT	Underlined: BamHI site
CDEP4658	AGGGTAAAGAGGAGAGTCGACTAAGGAGGATTAATATATGGTTAATATTATACT	Underlined: SalI site
CDEP4659	ACGACGGCCAGTGCCAAGCTTTATTCTTCCTTTCTAAAACAGTATCC	Underlined: HindIII
CDEP4660	ATATCTGGAAGCATCACTTCTAAAAGAGCTATTGTAATATCATTTTCTG	
CDEP4661	TTACAATAGCTCTTTTAGAAGTGATGCTTCCAGATATAAGTG	
CDEP4719	AATTAAACTGTAAATGGCCA **GCACTCATAGGTGAATTAGT** GTTTTAGAGCTAGAAATAGC	Underlined: MscI; Bold: sgRNA-cd630_27680-1
CDEP4720	AATTAAACTGTAAATGGCCA **TTACATTTAAAAAATCACAC** GTTTTAGAGCTAGAAATAGC	Underlined: MscI; Bold: sgRNA-cd630_27680-2
CDEP4722	CGATAGTTATGAAGTGAGCTCGAAAGGATTAGGTATTTGAGAT	Underlined: SacI site
CDEP4723	TTATTAAAACTTATAGGATCCCTATGTAAATCAATATCTATTAttaTTGTAAAACACGT	Underlined: BamHI site
CDEP4937	CGATAGTTATGAAGTGAGCTCGGGAACAAAGTTAAAAAATTAAGT	
CDEP4938	AAGTTTTATTAAAACTTATAGGATCGCTTTTTATTTCttaTTTCATAAATCCTAGT	
CDEP4939	AATTAAACTGTAAATGGCCA **GCATTTTTTGCTTTTGCAAA** GTTTTAGAGCTAGAAATAGC	Underlined: MscI; Bold: sgRNA-cwpV
CDEP5110	AATTAAACTGTAAATGGCCA **TCCTGAGGAGAATAATCTTT** GTTTTAGAGCTAGAAATAGC	Underlined: MscI; Bold: sgRNA-25040-1
CDEP5111	AATTAAACTGTAAATGGCCA **CTCGCTTCACTGCTTTCCTG** GTTTTAGAGCTAGAAATAGC	Underlined: MscI; Bold: sgRNA-25040-2
CDEP5112	AATTAAACTGTAAATGGCCA **TATAAATACTACTAAAATAA** GTTTTAGAGCTAGAAATAGC	Underlined: MscI; Bold: sgRNA-36010-1
CDEP5375	CAGGCTTCTTATTTTTATGGTACCGTATTGGTCATGTTGATGGATTTG	Underlined: KpnI site
CDEP5376	TCTCCTTTACTGCAGGAGCTCTTATTTCATATATACTACCTCTCTTTTCTTTAG	Underlined: SacI site
CDEP5394	CAGGCTTCTTATTTTTATGGTACCCAATCCTTCTTATTTACTTAAAATCCTG	Underlined: KpnI site
CDEP5395	TCTCCTTTACTGCAGGAGCTCTTATTTCATATTTAGTTCTCCTTTTTAAATTTATTG	Underlined: SacI site
CDEP5396	CAGGCTTCTTATTTTTATGGTACCGAACTTTATATCATATGTAAAAATTAAGGGAG	Underlined: KpnI site
CDEP5397	TCTCCTTTACTGCAGGAGCTCTTACTTCATAACAACATCCTCCAAG	Underlined: SacI site
CDEP5398	caGGCTTCTTATTTTTATGGTACCTACTTTACTAAGCTACTTCCACTC	Underlined: KpnI site
CDEP5399	TCTCCTTTACTGCAGGAGCTCTTATTCcatGATTTCCTCCCCAAAAG	Underlined: SacI site
CDEP5420	CAGGCTTCTTATTTTTATGGTACCGACTTTTAAATTTCTTATCATAGGATTTTAAG	Underlined: KpnI site
CDEP5421	TCTCCTTTACTGCAGGAGCTCTTATTTCATATCTTACCTCCTAAATTAAATG	Underlined: SacI site
CDEP5485	AGGAATTAGGGATGTAATAAGCGGATCCTTATTTATATAATTCATCCATAC	
CDEP5486	ACGACGGCCAGTGCCAAGCTGGTACCGACTTTTAAATTTCTTATC	
CDEP5641	AATTAAACTGTAAATGGCCA**TACCATCATTGCACTTATAA** GTTTTAGAGCTAGAAATAGC	Underlined: MscI; Bold: sgRNA-05490
CDEP5642	AATTAAACTGTAAATGGCCA**GTTATAAAAACCTTATCTAT** GTTTTAGAGCTAGAAATAGC	Underlined: MscI; Bold: sgRNA-27940
CDEP5643	AATTAAACTGTAAATGGCCA **GCTCCTTTAGCCTTTGCAAA** GTTTTAGAGCTAGAAATAGC	Underlined: MscI; Bold: sgRNA-27860
CDEP5644	AATTAAACTGTAAATGGCCA **GTAGTTTCTGCAGCAAAAAC** GTTTTAGAGCTAGAAATAGC	Underlined: MscI; Bold: sgRNA-27910

Oligo	Sequence	Relevant features
CDEP5645	AATTAAACTGTAAATGGCCA **TCAGTAGCAAATGCCATTGT** GTTTTAGAGCTAGAAATAGC	Underlined: MscI; Bold: sgRNA-10360
CDEP5664	CAGGCTTCTTATTTTTATGGTACCCTAAATTTACATACATTTTGAGCTC	Underlined: KpnI site
CDEP5665	TCTCCTTTACTGCAGGAGCTCTTATACCATAATTACAGCATTCCCC	Underlined: SacI site
CDEP5705	AATTAAACTGTAAATGGCCA **AATTTACATCCATATAATCA** GTTTTAGAGCTAGAAATAGC	Underlined: MscI; Bold: sgRNA-07380-1
CDEP5706	AATTAAACTGTAAATGGCCA **ATTGCATTTTTTTCTGCTTT** GTTTTAGAGCTAGAAATAGC	Underlined: MscI; Bold: sgRNA-07390-1
CDEP5707	AATTAAACTGTAAATGGCCA **AATTATCTTGCAATTTTTCT** GTTTTAGAGCTAGAAATAGC	Underlined: MscI; Bold: sgRNA-07380-2
CDEP5708	AATTAAACTGTAAATGGCCA **AAAGTAAAAAAATTTAAAAG** GTTTTAGAGCTAGAAATAGC	Underlined: MscI; Bold: sgRNA-07390-2
CDEP5715	CGATAGTTATGAAGTGAGCTCAAGGAGGAACTAAATatgAAAAAAAGATTAATAATAATG	Underlined: SacI site
CDEP5717	CGATAGTTATGAAGTGAGCTCAAGGAGGGGTAAGATatgAAAGTAAAAAAATTTAAAAG	Underlined: SacI site
CDEP5718	TCTATTTAAAGTTTTATTAAAACTTATAGGATCCttaTTTTTCAAGCTCATCAAAGTAAT	Underlined: BamHI site
CDEP5719	TTCATATCTTACCCCTCCTTTTTAAAGTTATTATTTCATAAGTTTATCG	
CDEP5720	TAATAACTTTAAAAAGGAGGGGTAAGATATGAAAGTAAAAAAATTTAAAAG	
CDEP5721	TTCATATTTAGTTCCTCCTTTCTTTAAGCATTATTTTTCAAGCTC	
CDEP5722	TAATGCTTAAAGAAAGGAGGAACTAAATATGAAAAAAAGATTAATAATAATGATATTG	
CDEP5725	TTATTAAAACTTATAGGATCCttaTTTCATAAGTTTATCGTTTTCCAT	Underlined: BamHI site
CDEP5726	TTATTAAAACTTATAGGATCCttaTTTCATAAGTTTATCGTTTTCC	Underlined: BamHI site
P2405	GCCGCTGTATCCATATGACC	
P2406	AATTAGGATAGGAAAACGATAG	
P2408	GAGGAGAGTCGACGCATGG	Underlined: SalI site
P2411	GTGGCTAGCCTGGCATCTTTTTATTTAGGGATTTCTCAC	Underlined: NheI site

Table S3. Wal regulon genes tested for effects on induction of the regulon

Gene locus	Gene name	Description	Wal-ON or Wal-OFF	Rationale	Overexpression	CRISPRi
cd630_05140	*cwpV*	Cell wall binding protein	OFF	Most highly induced, S-layer associated	pCE791	pCE789
cd630_05490		Hypothetical protein	ON	Highly induced		pIA102
cd630_07380		Hypothetical protein	ON	Highly induced; lysozyme regulon[1]	pIA112, pIA114, pIA115	pIA110, pIA111
cd630_07390		Hypothetical protein	ON	Most highly induced, lysozyme regulon[1]	pIA113, pIA114, pIA115	pIA108, pIA109
cd630_10360	*cwp17*	Cell wall binding protein	ON	Moderately induced, S-layer associated (amidase)		pIA106
cd630_15220	*pgdA*	PG deacetylase	ON	Highly induced, PG related		pCE655
cd630_25040		D-alanyl-D-alanine carboxypeptidase	OFF	Weakly repressed, PG associated[2]		pIA79, pIA80
cd630_27680		Cell-wall hydrolase	ON	Moderately repressed, hydrolase	pCE741	pCE744
cd630_27860	*cwp5*	Cell wall binding protein	ON	Moderately induced, S-layer associated		pIA104
cd630_27910	*cwp2*	Cell wall binding protein	ON	Moderately induced, S-layer associated, putat. WalR binding site		pIA105
cd630_27940	*cwp12*	Cell wall binding protein	ON	Moderately induced, S-layer associated, putat. WalR binding site		pIA103
cd630_28540	*dltD*	D-alanine transferase	OFF	Moderately repressed, cell wall associated, putative WalR binding site		pCE738
cd630_36010		D-alanyl-D-alanine carboxypeptidase	ON	Moderately induced, PG related		pIA81

[1]: (43)
[2]: See Table S1

Table S4. Putative WalR binding sites

Gene	Name	Description	Orientation[1]	Search[2]	Wal-ON or Wal-OFF
cd630_08670		TGTTTA-GTAAT-GTGAAT	Forward	C	ON, up
cd630_07400		TGTAAA-GCCTC-CTTAAA	Reverse	C	ON, up
cd630_27940	*cwp12*	TGTGAT-TTTCA-TTTTAT	Reverse	C	ON, up
cd630_03910	*asnB*	TGTTAT-AACAC-TGATAA	Forward	B	ON, up
cd630_14960		TGTTAT-AGAAT-CGGAAG	Reverse	C	ON, up
cd630_27910	*cwp2*	AGTAAC-AAAAT-TGTAAT	Reverse	B	ON, up
cd630_28620		TGTTTT-GCCTT-TTTAGT	Reverse	C	ON, up
cd630_20580		TGTAAA-TAAAC-TGTAAA	Reverse	A	ON, up
cd630_07410	*glpK1*	TGTAAA-GCCTC-CTTAAA	Forward	C	ON, up
cd630_26810		TGTCAA-ACACA-CTGAAA	Forward	C	ON, up
cd630_22280		TGTTTT-TTGTT-TTTTAT	Reverse	C	ON, up
cd630_18070		TGTAAT-AAAAT-TGAAAC	Reverse	B	ON, up
cd630_30360		TGTAAA-ACAAT-TTGTAA	Reverse	B,C	ON, down
cd630_10540	*bcd2*	TGTCAT-AATTG-GTTAGT<br>TGTTGC-CGAGA-TTTTAA	Forward<br>Forward	C<br>C	ON, down
cd630_27680		TGTAAT-CTTTT-TGTAAT<br>TGTAAT-CTTTT-TGTAAT	Reverse<br>Reverse	A<br>A	ON, down
cd630_22600		TGGTAA-AATTT-TGTTAA	Forward	B	ON, down
cd630_17290		TGTAAA-AAATA-TGTAAA	Reverse	A	Off, up
cd630_23920		TGTTTA-AACTA-TTGAAA	Reverse	C	Off, up
cd630_32630	*pstC*	TGTATT-CAAAA-TTTAAT	Reverse	C	Off, up
cd630_28540	*dltD*[3]	TGTTAC-AAAAT-TGTAAA	Reverse	A	Off, down
cd630_05300		TGTTTA-AATAT-TGTAGG	Reverse	C	Off, down
cd630_28620		TGTTTT-GCCTT-TTTAGT	Reverse	C	Off, down

[1]:Orientation: Putative WalR binding site is in the same orientation as gene (forward) or in the opposite direction (reverse).
[2]:Search catergories: (A) TGTWAH-N5-TGTWAH (14), (B)) TGTWAH-N5-TGTWAH, one mismatch allowed, (C) TGTNDH-N5-BKBWRN (8).
[3]: Previously described as part of a tandem direct repeat sequence (49).

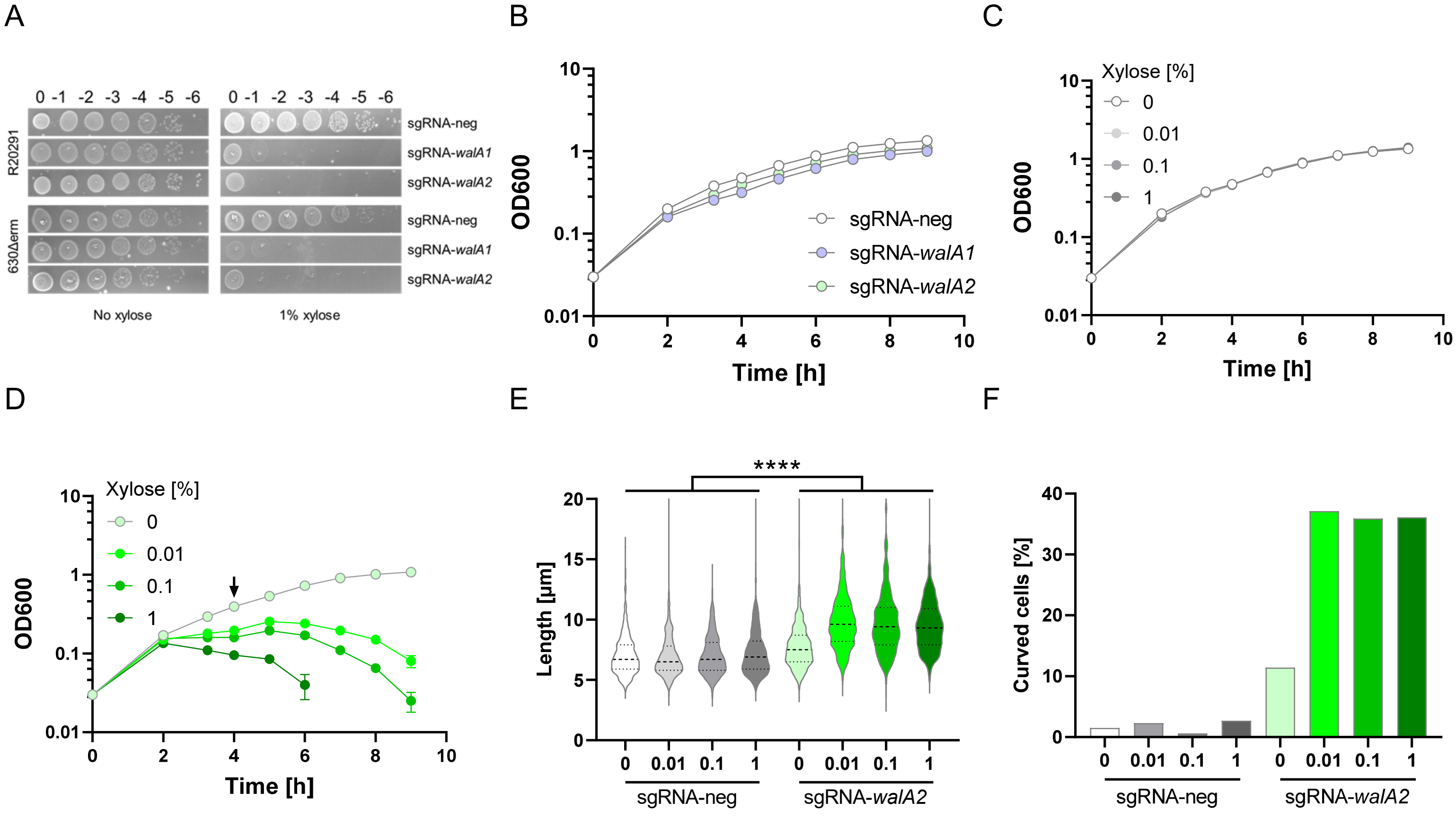

Fig. S1. CRISPRi silencing of the *wal* operon. (A) Plasmid-based CRISPRi silencing of the *wal* operon causes a strong viability defect. Serial dilutions of overnight cultures were spotted onto TY-Thi plates with or without 1% xylose to induce the expression of dCas9. Plates were photographed after incubation overnight. The strains shown are exconjugants of R20291 or 630Δerm harboring the following CRISPRi plasmids: pIA34 (sgRNA-neg, negative control), pIA50 (sgRNA-*walA1*) or pIA51 (sgRNA-*walA2*). (B-F). Silencing of the *wal* operon with a chromosomal copy of the CRISPRi machinery causes growth and morphology defects. (B) Growth curves of sgRNA-neg (UM554), sgRNA-*walA1* (UM555) and sgRNA-*walA2* (UM556) in TY, no xylose. (C) Growth curve of sgRNA-neg (UM554) in TY with 0, 0.01, 0.1 or 1% xylose. (D) Growth curves of sgRNA-*walA2* (UM556) in TY with 0, 0.01, 0.1 or 1% xylose. (E) Cell length and (F) percent cells with curved contours based on measurements of about 500 cells per condition. Samples were taken 4h after subculture (arrow in D). The sgRNA-*walA2* strain is longer than the sgRNA-neg control in all pairwise comparisons as determined by a T-test, $p<0.0001$. Data shown are representative of at least 2 independent experiments.

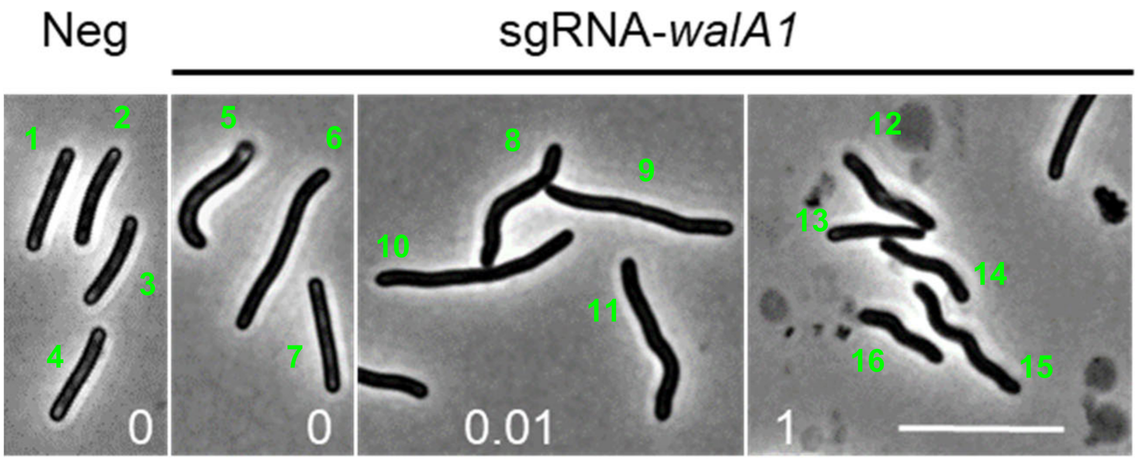

Cell	Sinuosity
1	1.001
2	1.008
3	1.010
4	1.003
5	1.183
6	1.011
7	1.001
8	1.066

Cell	Sinuosity
9	1.009
10	1.032
11	1.046
12	1.020
13	1.008
14	1.039
15	1.039
16	1.032

Fig. S2. Sinuosity examples. Cells from Fig 2C are numbered and their measured sinuosity reported in the table below. Orange highlighting indicates sinuosity values above 1.03, i.e. cells that were counted as "curved" in Fig. 2E. Strains shown are sgRNA-neg (UM554) and sgRNA-*walA1* (UM555). Numbers at the bottom of each image are the % xylose used for induction of *dCas9*. Bar = 10 μm.

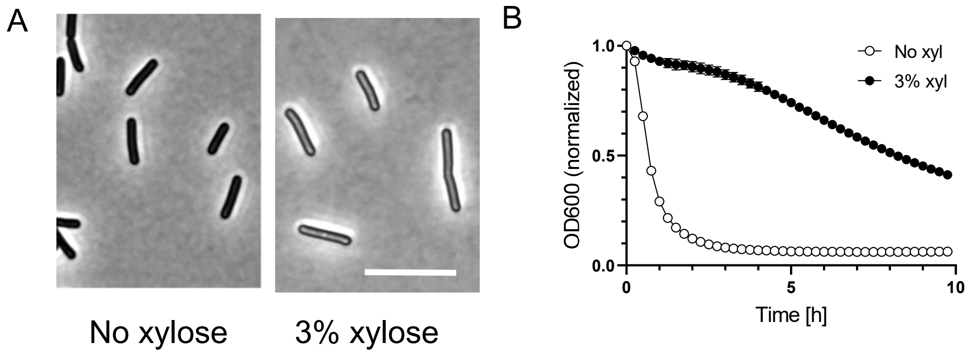

Fig. S3. Overexpression of *walR* from a multi-copy plasmid alters morphology and slows autolysis in R20291. (A) Phase contrast microscopy and (B) Lysis assay of R20291 harboring pCE691 (P_{xyl}::*walR).* Duplicate cultures were grown in TY-Thi to an OD_{600} of 0.2, at which time one was induced with 3% xylose (green arrow). Both cultures were harvested after 3h. Bar = 10 µm. Data shown are representative of at least 3 experiments. Error bars in (B) depict SD of 3 technical replicates but are mostly smaller than the symbols.

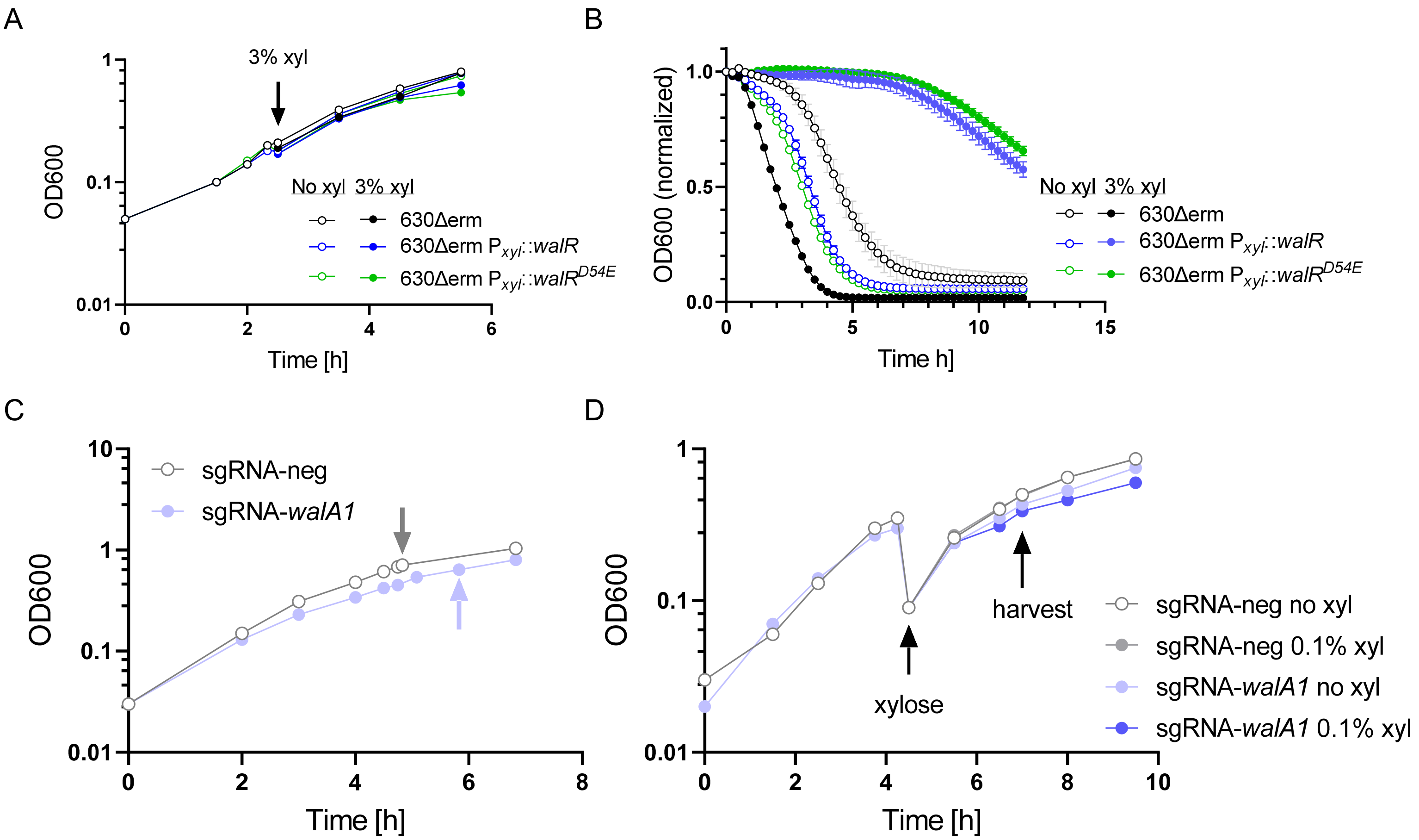

Fig. S4. Culture growth for RNA-seq experiments. (A, B) P_{xyl}::*walR* strains for RNA-seq under Wal-ON conditions. (A) Growth curves. (B) Lysis assay. Error bars in (B) depict SD of 3 technical replicates but are mostly smaller than the symbols. Strain 630Δerm (UM275) and derivatives with P_{xyl}::*walR* (UM626) or P_{xyl}::*walR-D54E* (UM628) integrated at *pyrE* were grown overnight in TY-Thi. These cultures were diluted in duplicate into the same medium to a starting OD_{600} of 0.05, grown to OD_{600} ~0.2, one culture set was induced with 3% xylose, and all cultures were harvested 3h later. RNA was purified from the induced cultures only. The non-induced cultures were included for growth and lysis phenotype comparison. (C, D) sgRNA-*walA1* strain for RNA-seq under Wal-OFF conditions. (C) Growth curves in TY without xylose. Overnight cultures of the CRISPRi strain UM555 (sgRNA-*walA1*) and the matched control UM554 (sgRNA-neg) were subcultured to OD_{600} of 0.03 and harvested at OD_{600} ~0.7 (arrows). (D) Growth curves of the same strains in TY with low xylose induction. Overnight cultures grown in TY were subcultured into TY to OD_{600} of 0.03 and grown to OD_{600} of 0.3. These cultures were then diluted in duplicate to OD_{600} = 0.1 and one culture set was induced with 0.1% xylose. Cells were harvested after 2.5h of induction. RNA was purified from the induced cultures only. The non-induced cultures were included for growth comparison.

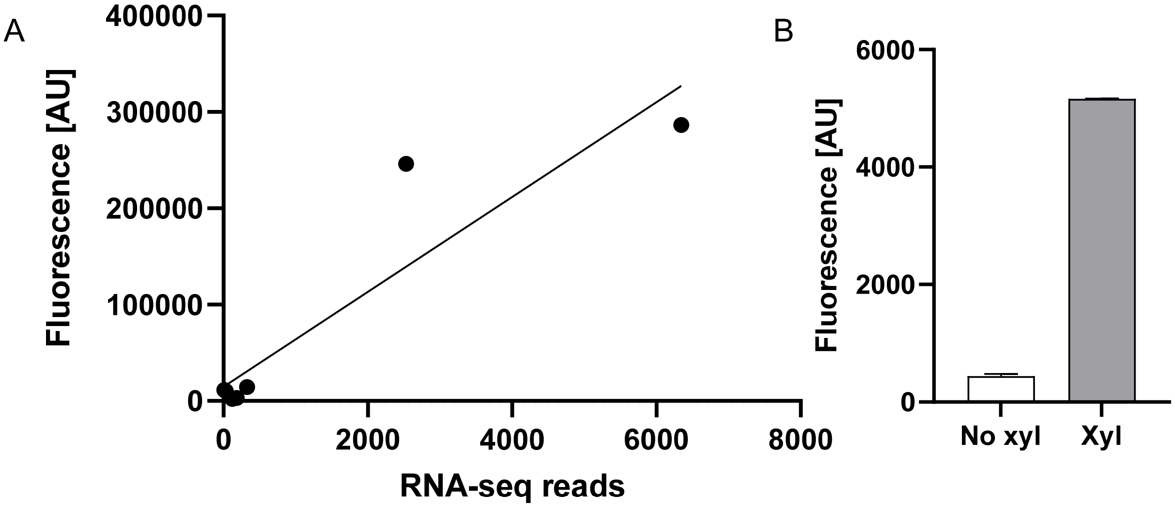

Fig. S5. RNA-seq follow-up. (A) Baseline expression of *rfp* reporter plasmids correlates with baseline RNA-seq reads. 630Δerm exconjugants harboring plasmids with transcriptional fusions of *rfp* to several WalR regulon promoters were grown in TY-Thi and red fluorescence was measured by flow cytometery. RNA-seq reads are from strain UM554 grown in TY without xylose. The genes shown are the same as Fig. 6: *pgdA, dltD, cd630_0738, cd630_0739, cd630_08670, cd630_ 53000, cd630_28620.* (B) Chromosomal P_{cd630_0739}*::rfp* responds to overexpression of walR. Strain UM926 (630Δerm P_{cd630_0739}*::rfp*) harboring plasmid pCE691 (P_{xyl}::walR) was subcultured to OD_{600} of 0.05, grown to 0.35 and induced with 3% xylose for 3 h. Fluorescence was measured by flow cytometry.

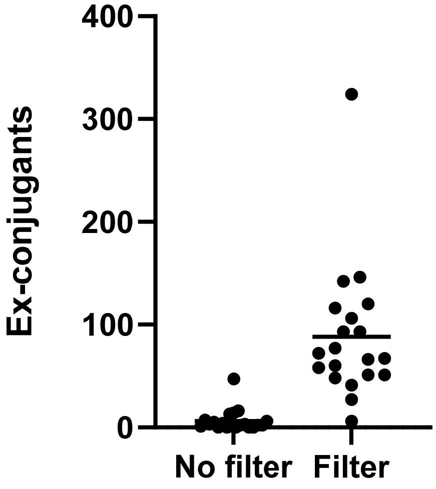

Fig. S6. Mating on filters improves conjugation efficiency. Twenty different CRISPRi plasmids were conjugated from *E. coli* into R20291 by mating directly on BHI plates or on cellulose filters. Conjugation success reported as number of ex-conjugants increased by more than 10-fold with the use of filters.
